# Xuanfei Baidu decoction alleviates intestinal inflammation and modulates microbiota distribution by suppressing IMD/NF-κB and JNK signaling pathways

**DOI:** 10.3389/fmicb.2026.1853715

**Published:** 2026-06-10

**Authors:** Yidong Xu, Xinyi Li, Yaodong Miao, Xiaoxi Liu, Xiya Zang, Qinghao Meng, Ying Li, Fengwen Yang, Zhiguang Yuchi, Yiwen Wang

**Affiliations:** 1 Faculty of Medicine, School of Pharmaceutical Science and Technology, Tianjin University, Tianjin, China; 2 Second Affiliated Hospital of Tianjin University of Traditional Chinese Medicine, Tianjin, China; 3 First Teaching Hospital of Tianjin University of Traditional Chinese Medicine, Tianjin, China; 4 National Clinical Research Center for Chinese Medicine, Tianjin, China; 5 State Key Laboratory for the Creation of Modern Traditional Chinese Medicine, Evidence-Based Medicine Center, Tianjin University of Traditional Chinese Medicine, Tianjin, China

**Keywords:** COVID-19, intestinal flora, intestinal inflammation, JAK/STAT, JNK, NF-κB, Xuanfei Baidu decoction

## Abstract

**Objective:**

Xuanfei Baidu decoction (XFBD), one of China’s recommended prescriptions for COVID-19, has demonstrated clinical efficacy in mitigating gastrointestinal inflammation associated with SARS-CoV-2 infection, yet its exact therapeutic mechanism remains incompletely understood, necessitating further *in vivo* investigation. In this study, we utilized a fruit fly (*Drosophila melanogaster*) model of intestinal inflammation to assess the therapeutic potential and underlying mechanisms of XFBD in ameliorating intestinal inflammation.

**Methods:**

We induced intestinal inflammation in *D. melanogaster* by dextran sulfate sodium (DSS) administration. We examined XFBD’s protective effects on *D. melanogaster*’s intestine and explored changes in the IMD and JNK-JAK/STAT signaling pathways. Furthermore, we analyzed the intestinal microbiota through 16S rRNA gene sequencing.

**Results:**

XFBD effectively mitigated DSS-induced intestinal inflammation, leading to a significant extension of the flies’ lifespan. It enhanced the integrity of intestinal barrier while reducing reactive oxygen species (ROS) levels in intestine. Additionally, XFBD restored the disrupted intestinal microbiota by regulating immune homeostasis, notably inhibiting the IMD, Toll, and JNKJAK/STAT pathways. Furthermore, the reintroduction of symbiotic microbiota partially alleviated the DSS-induced dysregulation.

**Conclusion:**

In conclusion, our study underscores the therapeutic potential of XFBD for intestinal inflammation, providing valuable insights into its key mechanisms and highlighting the pivotal role of symbiotic microbiota in anti-inflammatory responses and immune pathway regulation.

## Graphical abstract

**Figure:**
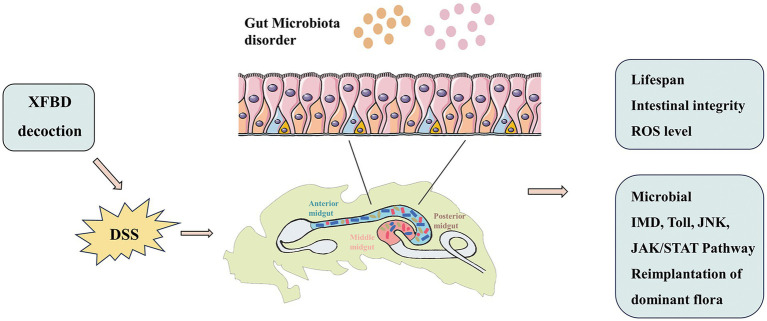

## 1 Introduction

Typically, COVID-19 patients primarily exhibit respiratory symptoms (e.g., fever, dry cough, and sore throat) (Nile et al., 2020), and exhibit prominent gastrointestinal manifestations including diarrhea, anorexia, and nausea (Aiyegbusi et al., 2021).

In the gastrointestinal tract, inflammatory reactions can impact the secretion of inflammatory cytokines and alter the composition of the microbiota (Man, 2018). The disruption of the gut flora can result in an imbalance of antiviral immunity, thereby exacerbating gastrointestinal and lung diseases (Al-Momani et al., 2024; Yan et al., 2020). Clinical studies have demonstrated that the disturbance of the gut microbiota in COVID-19 patients may lead to the proliferation of opportunistic pathogens in the gut, subsequently causing inflammation, diarrhea, and other symptoms (Velikova et al., 2021; Wang et al., 2025). Furthermore, patients with post-acute COVID-19 syndrome (PACS) have exhibited a significant reduction in both the diversity and richness of their gut microbiota compared to healthy individuals (Liu et al., 2022).

XFBD is one of the “three drugs and three prescriptions” approach proposed in China to combat COVID-19. It consists of 13 Traditional Chinese Medicine (TCM), including Ephedrae Herba, Polygoni Cuspidati Rhizoma et Radix, Glycyrrhizae Radix et Rhizoma, Semen Coicis, Gypsum Fibrosum, Rhizoma Areactylodis lanceae, Artemisiae Annuae Herba, Pogostemon patchouli, Descurainiae Semen, European Verbena Herb, *Phragmites communis* Trin, Tomentose Pummelo Peel, and Armeniacae Semen Amarum (Wang et al., 2020). Recent research indicates that XFBD offers advantages in the antiviral treatment and addressing intestinal flora disorders due to its multifaceted composition, multiple targets, and diverse therapeutic pathways (Zhang et al., 2023). Utilizing network pharmacology, molecular docking, and molecular dynamics analysis, XFBD’s effects are proposed to result from interactions between its active components and core target proteins, potentially influencing key factors like IL4, IL1B, and JUN. This modulation may regulate signaling pathways related to TNF, IL-17, and Th17 cell differentiation. Additionally, I-SPD and Pachypodol can reduce the inflammatory response and cell apoptosis by inhibiting the activation of NLRP3, and they can also reduce the production of inflammatory mediators (Xiong et al., 2022). Ultimately, this action stabilizes the balance of intestinal flora and microbial metabolism while enhancing the antioxidant, anti-inflammatory, and immunomodulatory activities of XFBD (Zhao et al., 2023).

*Drosophila melanogaster* (fruit fly) is a classic model organism in genetics and molecular biology, boasting prominent advantages over mammals that facilitate efficient and rigorous research on intestinal inflammation. These include a short lifecycle, rapid reproduction (enabling rapid experimental turnover and high-throughput phenotypic analyses), low experimental cost, and diverse, sophisticated genetic tools for precise genetic manipulation (Zhang et al., 2024). Notably, 75% of human disease-causing genes have functional homologs in fruit flies (Pandey and Nichols, 2011), making them widely used to model human disorders—including intestinal disorders—for pathological research and drug development, especially screening natural products in Traditional Chinese Medicine (TCM) and elucidating their therapeutic mechanisms. As a common inducer of intestinal inflammation, oral DSS administration triggers robust inflammatory phenotypes in *D. melanogaster*, including shortened lifespan, impaired locomotion and excretion, reduced intestinal length and integrity, disrupted acid–base homeostasis in the copper cell region, elevated intestinal stem cell counts, and altered gut microbiota diversity (Amcheslavsky et al., 2009; Keshav et al., 2022; Yan et al., 2023); these symptoms closely mirror those of DSS-induced murine intestinal inflammation models (Xinyi Li et al., 2025), effectively recapitulating the core pathological phenotypes of mammalian and human intestinal inflammation to ensure the translational relevance of the findings. Thus, the fruit fly model represents a valuable alternative to mammalian systems, accelerating research progress, cutting costs, and resolving ethical concerns. Indeed, this DSS-induced *Drosophila* intestinal inflammation and dysbiosis model has been extensively utilized to dissect the therapeutic efficacy and mechanistic underpinnings of numerous TCM herbs and natural products in ameliorating intestinal inflammation (He et al., 2025; Li et al., 2024; Xiu et al., 2025).

In this study, we developed a fruit fly model of intestinal inflammation through DSS-feeding to simulate conditions characterized by gut microbiota dysbiosis. Our aim was to examine the exact nature of microbial imbalances within the intestinal flora and to elucidate the specific mechanisms responsible for XFBD’s protective effects on the intestine.

## 2 Methods and materials

### 2.1 The fly stocks and culture conditions

The wild type fruit fly strain in this study was Dijon 2000, collected from France by Dr. Jean-Francois Ferveur (Grillet et al., 2012). ESG-Gal4, UAS-GFP (TB00093) and upd3-Gal4, UAS-GFP (THU0198) flies were obtained from Tsinghua Fly Center (THFC). The 10 × STAT GFP, Myo1A-Gal4/tub-Gal80ts/UAS-GFP, and UAS-Relish/GD II fly lines were donated by Professor Song’s lab at Wuhan University (Ding et al., 2021). All fly lines were maintained on a standard cornmeal diet (Li et al., 2023). Flies were reared at 25 ± 1 °C or 28 ± 1 °C and 70% humidity, under a 12/12-h night/dark cycle.

### 2.2 Chemicals

XFBD was provided by Tianjin Modern TCM Innovation Center (TRT, 200302). The specific preparation method can be found in the article published by Yan et al. (2021). And the HPLC-based chemo profile of XFBD was included in the supplementary data.

### 2.3 Liquid chromatography quadrupole time-of-flight mass spectrometry (LC-Q-TOF-MS) analysis of XFBD

An ACQUITY ultra-performance liquid chromatography (UPLC) system (Waters, USA), coupled with a triple time-of-flight (TOF) 5,600+ MS (AB SCIEX, USA) and equipped with an electrospray ionization (ESI) source, was used for chemical identification. Samples were separated on a Waters ACQUITY UPLC HSS T3 column (150 mm × 2.1 mm, i.d., 1.8 μm) at a flow rate of 0.3 mL/min and a column temperature of 50 °C. The injection volume was 10 μL, and the detection wavelength was 254 nm. The mobile phases were as follows: 0.1% formic acid–water as an aqueous mobile phase (A) and 0.1% formic acid–acetonitrile as an organic mobile phase (B). The linear gradient elution was optimized as follows: 0–2 min, 0% B; 2–25 min, 0–30% B; 25–35 min, 30–95% B; 35–37 min, 95% B. An MS analysis was performed in both positive and negative modes under the following parameters: scan range, mass-to-charge ratio (m/z) scan ranges 90–1,500 Da (positive) 100–2000 Da (negative); ion source GS1, 55 psi (1 psi = 6.89 kPa); ion source GS2, 55 psi; curation gas (CUR), 35 psi; temperature, 600 °C for positive-ion ESI (ESI+) and 550 °C for negative-ion ESI (ESI-); ion spray (IS) voltage, −4.5 kV for ESI-and 5.5 kV for ESI+; declustering potential (DP), 100 V; and collision energy (CE), 10 V.

### 2.4 Establishment of DSS induced gut inflammation model and XFBD treatment

Three days old (post eclosion) female flies were transferred to vials containing five filter papers soaked with 400 μL one of the following solutions to feeding: 5% sucrose (control group, ND), 5% sucrose and 3% DSS (DSS group, DSS, aladdin^®^, Shanghai, China, 500,000 molecular weight), or 5% sucrose, 3% DSS and XFBD at various concentrations (XFBD groups, DSS + XFBD). The filter papers were replaced with fresh ones every 24 h to maintain the treatment conditions. For the lifespan test, fruit flies were continuously exposed to DSS until death. For other physiological and molecular biology experiments, fruit flies were sampled and analyzed after 8 days of exposure to DSS or drug treatment. 10 g XFBD were dissolved in 50 mL ultrapure water, following diluted three times with ultrapure water, then centrifuged at 10000 rpm, 20 min. The supernatant was taken for LC-Q-TOF-MS analysis.

### 2.5 Lifespan assay

Twenty female flies were placed in each vial containing five filter papers soaked with the 400 μL corresponding solutions, with a total of five vials per experimental group. The date of mortality for each individual fly in each vial was documented until the demise of all flies in the group.

### 2.6 Smurf assay

After 8 days of treatment, twenty female flies were removed from their vials, subjected to 2-h fast, and subsequently transferred to fresh vials containing filter papers soaked with a Smurf dye solution (2.5 g Erioglaucine disodium salt dissolved in 100 mL of 5% sucrose solution) (Lyles et al., 2021) with a total of five vials per experimental group. The intestinal integrity of flies in each group was assessed at both 4 h and 24 h post-exposure to the Smurf dye solution. Flies without intestinal integrity, exhibited dye staining throughout their entire abdomen, were classified as “leaky gut.”

### 2.7 Selective medium spreading

Take distinct groups of twenty fruit flies and dissect their intestines in high-pressure sterilized 1× PBS solution. Homogenize the intestine in 200 μL of 1× PBS sterilized under high pressure and centrifuge at 1000 g for 1 min. Spread the culture medium using the supernatant and incubate it overnight at 37 °C. LB medium (Sangon Biotech, Shanghai, China, A507003), *Acetobacter,* and *Lactobacillus* (Qingdao Hope Biotechnology, Shandong, China, HB8879 and HB8636) selective mediums were prepared according to the manufacturer’s instructions. Calculate the relative abundance of bacteria from the colonies grown on the culture medium.

### 2.8 Bacterium replantation

Monoclonal isolates of *Acetobacter* and *Lactobacillus* were obtained from fly intestines in a previous study (Meng et al., 2024). Their taxonomic identities were confirmed via 16S rRNA gene sequencing, and the corresponding 16S rRNA gene sequences have been deposited in the NCBI GenBank database under accession numbers OQ519651 and OQ519650. The bacterial strains were cultured overnight in 10 mL of LB medium (Sangon Biotech, Shanghai, China, A507003) at 37 °C with agitation at 140 rpm. Following the overnight incubation, the bacteria were pelleted by centrifuged at 4000 rpm for 10 min. The bacterial pellets were re-suspended with 1 mL of DSS solution. The flies received a solution also containing *Acetobacter* or *Lactobacillus* every other day to ensure a continuous reintroduction of *Acetobacter* and *Lactobacillus* into their gut flora.

### 2.9 16S rRNA sequencing and analysis of intestinal microbiota

The fruit flies following 8 days of treatment were collected with fifty in each group and three parallel groups. Following surface sterilization, the flies were immediately frozen in liquid nitrogen, and stored in a refrigerator at −80 °C before sequencing. 16S rRNA extraction and sequencing were performed by BioMarker Technologies Company (Beijing, China). 16S results analysis was based on BMK Cloud.

Sequencing reads were quality-filtered, clustered into operational taxonomic units (OTUs) at 97% similarity, and taxonomically annotated using the SILVA database. Alpha diversity indices (Shannon, Simpson, ACE, and Chao1) were calculated with R v3.1.1 using the *picante* package (v1.8.2). Group-wise differences in alpha diversity were assessed using the Welch’s one-way ANOVA followed by Dunnett’s T3 multiple comparisons test. For beta diversity, PCA was performed based on OTU profiles using Python 2 (*scikit-learn* 0.17.1) to visualize overall microbial community dissimilarities between groups.

### 2.10 RT-qPCR

For one set of samples, whole-body from twenty flies that had undergone 8 days treatment with different solutions were used for RNA extraction. RNA extraction was performed using TriQuick reagent (Solarbio, Beijing, China, R1100) according to the manufacturer’s instructions. The concentration of RNA was measured using the NanoDrop™ One. The first cDNA chain was synthesized using Hifair^®^III 1st strand cDNA Synthesis SuperMix (Yeasen, Shanghai, China, 1141ES). RT-qPCR was performed on the Q2000A (LongGene^®^) real-time PCR instrument using Hieff^®^qPCR SYBR Green Master Mix (Yeasen, Shanghai, China, 11201ES). Relative expression levels were determined using the 2^−△△Ct^ method. Each group of experiments was conducted in parallel for four groups, with at least two replicates (primers are listed in Supplementary Table S2).

### 2.11 DHE staining

The method of DHE staining was based on a previous study (Evans et al., 2014). The intestines were dissected from live flies (*n* ≥ 8) in 1× PBS and incubated at 37 °C with 45 μM DHE probe (UElandy, Suzhou, China, D1008). After washing with PBS three times, and DAPI staining (1 μg/mL, Legagene, Beijing, China, DA0004), the intestine was rinsed three times with 1× PBS. The stained intestines were observed by NIKON fluorescence microscope (SMZ 1270). DHE intensity was quantified using ImageJ.

### 2.12 Statistical analysis

Data were presented as the mean ± standard error. The statistical analysis was conducted with GraphPad Prism 9 (Version No. 9.2.0, GraphPad Software, La Jolla, CA, USA). For qPCR analysis, the relative gene expression was calculated using the 2^−△△Ct^ method. For standard plate counting, intestinal ROS level measurement and fluorescent protein expression analysis, the data were log10-transformed to normalize the distribution and homogenize variances. Statistical analysis was performed using Welch’s one-way ANOVA followed by Dunnett’s T3 multiple comparisons test, with the DSS-induced model group set as the reference control. Statistical significance was defined as adjusted *p* < 0.05. ^*^Indicates *p* < 0.05; ^**^indicates *p* < 0.01; ^***^indicates *p* < 0.001. All mean gray values were quantified using ImageJ (v1.8.0; National Institutes of Health). Measurements represented the meaning of at least three biological replicates in all graphs.

## 3 Results

### 3.1 Characterization of the chemical constituents of XFBD by high-resolution MS

To further investigate the active components of XFBD, we analyzed its chemical composition using LC-Q-TOF-MS. Through accurate molecular weight determination, secondary mass spectrometry fragment ion analysis, referencing various medicinal herbs, and comparing with reference substances, we identified a total of ninety-one components. Among them, twenty-nine components were compared with the reference substance to confirm their structures. The active components identified in the analysis are consistent with 92% of the previously reported data (Zhao et al., 2023). However, there are still seven active components that have not been reported before, including Scoparone, Sinapinic acid, Ferulic acid, Isomer, Scopoletin, Quercetin 3-O-*β*-D-glucuronopyranoside, and Penduletin. Among the ninety-one identified components, there were thirty-two flavonoids, five monoterpenes, six sesquiterpenes, nine triterpenes, two alkaloids, eighteen phenolic acids and glycosides, three anthraquinones, and sixteen other compounds (Supplementary Figure S1, Table S1).

### 3.2 Administration of XFBD extends the lifespan and alleviates DSS-induced intestinal injury

**Figure 1:**
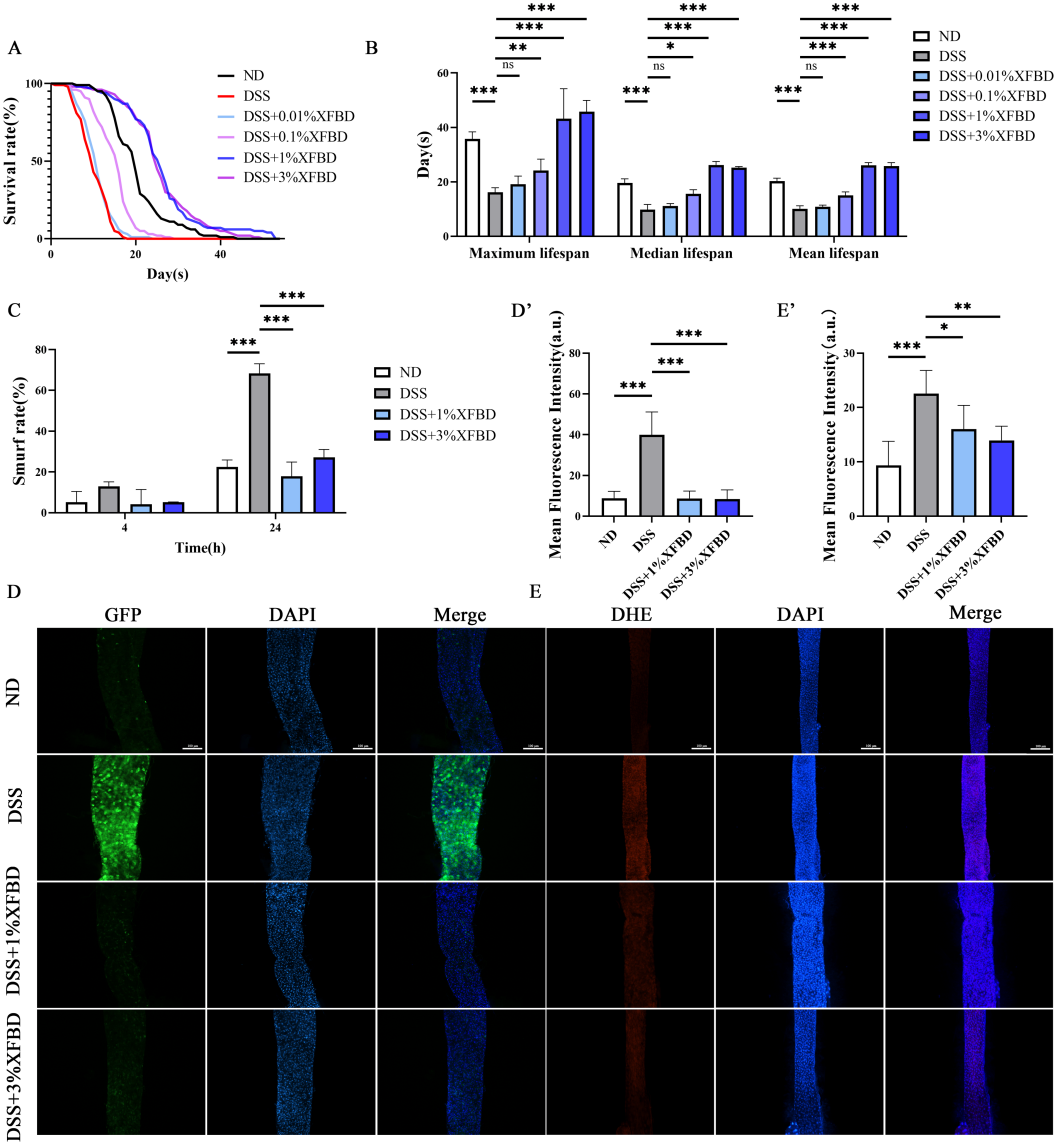
Effect of XFBD on the DSS induced intestinal inflammation in *Drosophila melanogaster*. **(A)** Survival curve (*n* = 20). **(B)** The maximum lifespan, median lifespan, and mean lifespan of *Drosophila melanogaster* in separate groups. **(C)** Effects of XFBD on the DSS induced intestinal integrity of *Drosophila melanogaster* (*n* = 20). **(D)** Fluorescence images of the midguts of ESG-GFP flies within different treatments (Green: ESG positive cells; Blue: DAPI staining, indicates the location of the nuclei. **(D’)** Mean fluorescence intensity of GFP. (E) DHE-staining. **(E’)** Mean fluorescence intensity of DHE (^*^indicates *p* < 0.05; ^**^indicates *p* < 0.01; ^***^indicates *p* < 0.001. Scale bar = 100 μm).

We examined XFBD on a fly model afflicted with DSS-induced intestinal inflammation. DSS-induced intestinal inflammation strongly reduced in the lifespan of fruit flies (Figures 1A,B). The XFBD treatment group effectively mitigated the premature mortality induced by DSS exposure in a dose-dependent manner (Figures 1A,B) confirming XFBD’s potent therapeutic efficacy. Notably, under DSS-induced intestinal disorder conditions, treatment with high XFBD concentrations (1 and 3%) extended the lifespan of flies beyond that of the control group under DSS-induced intestinal disorder. In contrast, the lowest concentration (0.01%) exerted no lifespan-extending effect, whereas the 0.1% concentration prolonged fly survival compared with the disease model group, though this effect did not reach the level observed in the healthy control group. Consequently, we selected these two dosages for subsequent experiments.

Besides a marked reduction in lifespan, intestinal inflammation triggers a series of physiological perturbations, including impaired intestinal barrier integrity, induced proliferation of intestinal stem cells (ISCs), and elevated reactive oxygen species (ROS) levels in the intestine (Yang et al., 2022).

We initially assessed the intestinal integrity of fruit flies at 4- and 24-h post-exposure by Smurf assay. The fruit flies exhibited a compromised intestinal barrier in the DSS treated model. 68% of the DSS-induced fruit flies experienced severe intestinal leakage at 24 h, which was three times the number in the control group. Both the 1% XFBD and 3% XFBD treatment groups demonstrated a remarkable preservation of intestinal integrity in response to DSS-induced damage at the 24-h time point (Figure 1C). In the 1% XFBD group, only 18% of fruit flies had intestinal leakage, and in the 3% XFBD group, only 27% experienced intestinal leakage.

Intestinal damage can stimulate proliferation and differentiation of ISCs, consequently facilitating the regeneration of intestinal epithelial cells (Yang et al., 2022). We utilized GFP labeling (ESG-Gal4, UAS-GFP) to assess the ISCs amplification within the flies’ intestine. The GFP signal intensity was notably higher in the guts of DSS-treated flies compared to the control group (Figures 1D,D’), corroborating the augmentation of ISC proliferation induced by DSS, consistent with previous reports (Du et al., 2020). Following the XFBD treatments, the GFP signal intensity exhibited a significant decrease, reaching levels comparable to those of the control group (Figures 1D,D’). This observation suggests that XFBD’s protective effect on the intestines of flies may potentially be attributed to decreased ISC amplification.

We also assessed ROS levels within the fruit fly intestine using DHE staining. Notably, DSS treatment led to a substantial increase in ROS levels within the gut of fruit flies. Administration of the 1 and 3% XFBD significantly reduced ROS levels compared to the DSS group (Figures 1E,E’). XFBD effectively eliminates excess ROS in fruit flies, which may contribute to its ability to counteract intestinal inflammation.

The above evidence proved that administration of XFBD can resist inflammation and prolong the lifespan of flies by clearing excess ROS and decreasing ISCs to maintain the homeostasis of the intestinal barrier.

### 3.3 XFBD treatment modulated the diversity of intestinal flora

We embarked on an investigation to ascertain whether the reduction of inflammation in fruit flies treated with DSS is correlated with an improved composition of the intestinal microbiota, as evaluated through 16S rRNA sequencing.

**Figure 2:**
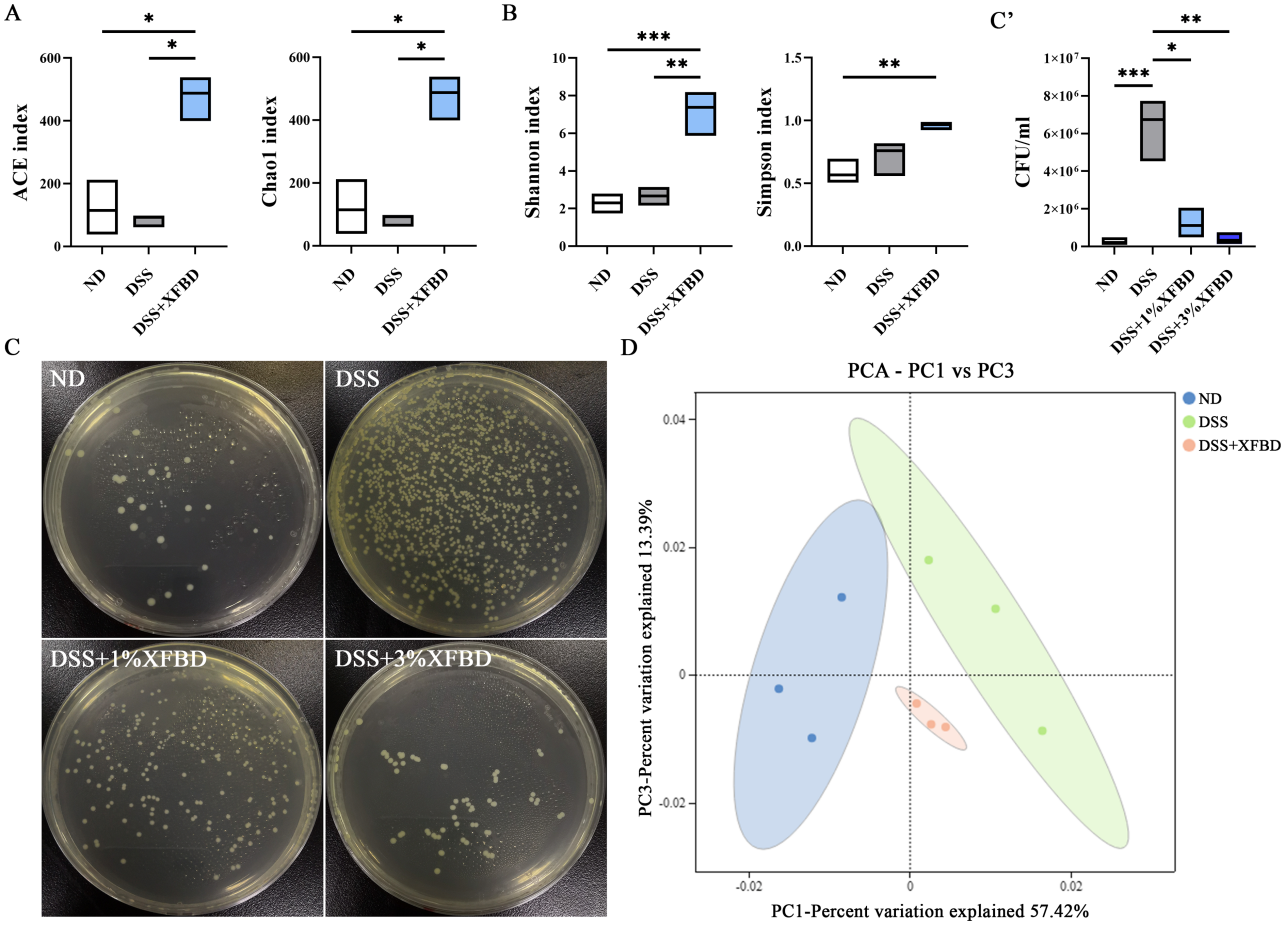
Intestinal microbiota analysis between the flies that received general, DSS group and XFBD group. **(A,B)** Alpha diversity analyzed by ACE, Chao 1, Simpson, and Shannon indexes. **(C)** LB medium cultivation to confirm the relative abundance of the intestinal microbiota of diverse groups. **(C′)** CFU number of LB plate. **(D)** PCA analysis of the β-diversity of gut microbiota (^*^indicates *p* < 0.05; ^**^indicates *p* < 0.01; ^***^indicates *p* < 0.001).

The Choa1 and ACE indices revealed a reduction in the number of species in the DSS-feeding group decreased when compared to the control group. In contrast, the XFBD-treated group exhibited a significant increase in species diversity compared to the DSS-induced group (Figure 2A). Although the Simpson and Shannon Indices did not exhibit significant changes following DSS administration, XFBD enhanced overall diversity, species diversity, and richness, surpassing both the control and DSS groups (Figures 2A,B). Moreover, the assessment of the gut microbiome’s culture aspects further substantiated these findings. DSS administration resulted in a significant increase in the total microbial counts (expressed as colony-forming units, CFU) of cultivable microorganisms, while XFBD exhibited a specific restorative effect on gut microbiota imbalance induced by DSS (Figures 2C,C’). Principal component analysis (PCA) revealed marked dissimilarity between the control and DSS groups, indicating a profound shift in microbiota composition due to DSS exposure. Importantly, XFBD treatment significantly altered the microbiota structure influenced by DSS. The PCA cluster for the XFBD treatment group was located between the control cluster and the DSS cluster. This result signifies its potential to partially restore gut microbial balance (Figure 2D).

### 3.4 XFBD improved DSS-induced microbiota dysbiosis at various taxonomic levels

**Figure 3:**
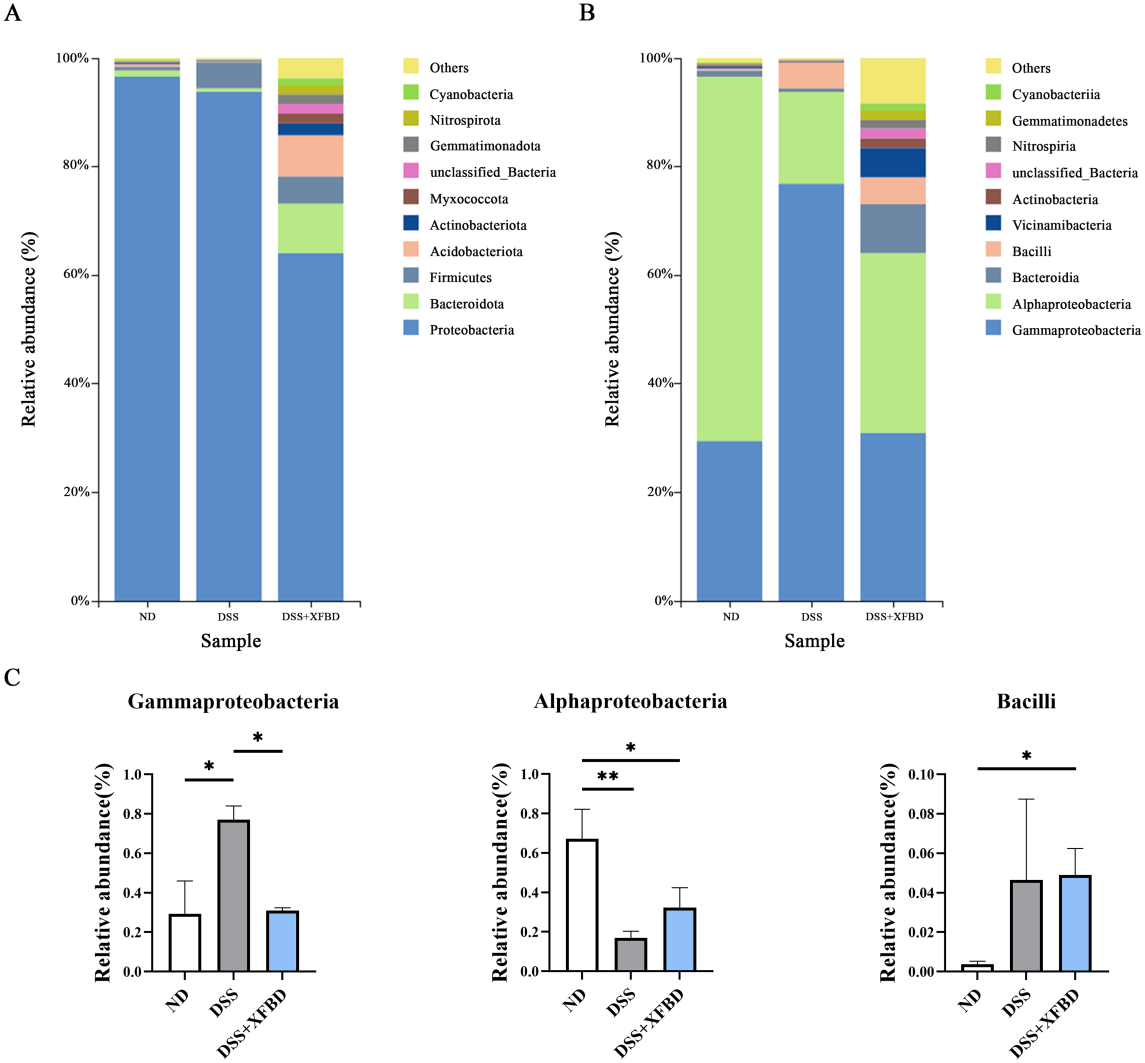
XFBD modulated the structure of the gut microbiota in DSS induced *Drosophila melanogaster*. **(A)** Phylum level. **(B)** Class level. **(C)** The relative abundance of *Gammaproteobacteria*, *Alphaproteobacteria*, and *Bacilli* at class level (^*^indicates *p* < 0.05; ^**^indicates *p* < 0.01; ^***^indicates *p* < 0.001).

We investigated the alterations in the intestinal microbiota composition at different taxonomic levels after DSS exposure, as well as after XFBD administration. At the phylum level, *Proteobacteria*, *Bacteroidota*, and *Firmicutes* were found to be the most abundant gut bacteria in flies (Figure 3A). Among these, *Proteobacteria* and *Firmicutes* are pivotal bacteria associated with aging and inflammation during the growth and development of fruit fly (Clark et al., 2015). An increase in relative abundance of *Firmicutes* was observed following DSS administration, while the relative abundance of *Proteobacteria* decreased significantly after treatment with XFBD. At the class level, we also noted variation in the relative abundance of *Gammaproteobacteria*, *Alphaproteobacteria*, and *Bacilli* after DSS exposure. The relative abundance of *Gammaproteobacteria*, a maker of the onset of intestinal injury (Clark et al., 2015), increased significantly after DSS treatment but returned to lower levels following XFBD administration (Figures 3B,C). Additionally, after DSS treatment, the relative abundance of *Alphaproteobacteria* decreased significantly, but XFBD reversed this decrease. *Alphaproteobacteria* encompasses various symbiotic bacterial species in fruit flies, with *Acetobacter* being a representative example. The restore of *Alphaproteobacteria* by XFBD treatment could be a significant factor contributing to the lifespan extension of fruit flies when subjected to DSS. The abundance of *Bacilli* significantly increased after DSS exposure. However, unlike *Gammaproteobacteria* and *Alphaproteobacteria*, there was no notable change in *Bacilli* abundance after XFBD feeding. This suggested a distinct difference between XFBD treatment and the control group, with a notable increase in microbial diversity, consistent with the results of PCA. This difference may contribute to the significant increase in the lifespan of fruit flies observed with XFBD treatment compared to the control group.

**Figure 4:**
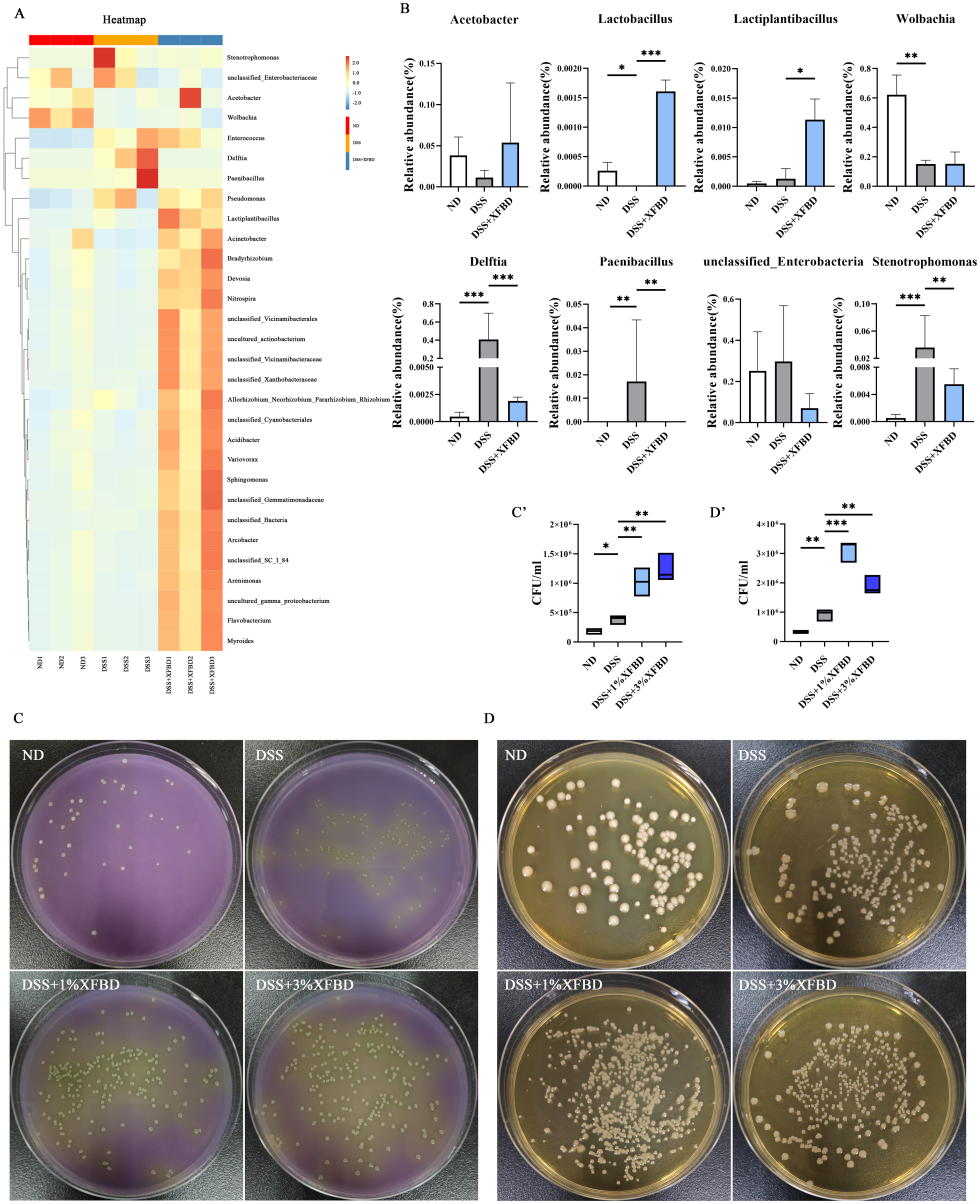
XFBD modulated gut microbiota at genus level. **(A)** The heatmap of taxa in three groups at genus level. **(B)** Effects of XFBD on the level of the representative bacteria at genus level. **(C)** *Acetobacter* selective medium cultivation to confirm the absolute abundance of the intestinal *Acetobacter* of diverse groups. **(C’)** CFU number of *Acetobacter* selective medium. **(D)** *Lactobacillus* selective medium cultivation to confirm the relative absolute of the intestinal *Lactobacillus* microbiota of distinct groups. (D′) CFU number of *Lactobacillus* selective medium (^*^indicates *p* < 0.05; ^**^indicates *p* < 0.01; ^***^indicates *p* < 0.001).

A heat map displayed differences in gut microbiota structure at the genus level (Figure 4A). Notably, *Acetobacter* level decreased after DSS treatment compared to the control group (Figure 4B). However, XFBD administration completely reversed the downregulation of *Acetobacter* induced by DSS feeding. Another symbiotic bacterium, *Lactobacillus*, also exhibited similar changes following DSS treatment (Figure 4B). The relative abundance of *Lactobacillus* nearly disappeared after DSS treatment, but the XFBD significantly increased its content (Figure 4B). In contrast, *Lactiplantibacillus* slightly increased after DSS treatment, and significantly increased after XFBD administration compared to the DSS group.

*Wolbachia*, a symbiotic bacterium distributed in the cells of arthropods, also significantly decreased after DSS treatment (Figure 4B). Conversely, the DSS group exhibited an increased relative abundance of *Delftia*, *Paenibacillus*, *unclassified_Enterobacteria* and *Stenotrophomonas.* Some of these species have been proven to be pathogenic bacteria, while others have been associated with opportunistic infections, and these bacteria reduced again after the administration of XFBD (Figure 4B).

To confirm alterations in the absolute abundance of intestinal microbes, we performed a culture-dependent quantitative analysis. Specifically, LB medium was used for the enumeration of general gut microbes, while selective media were employed for the quantification of *Acetobacter* and *Lactobacillus, respectively.* Compared to the control group, CFUs of the two dominate symbiotic bacteria, *Lactobacillus* and *Acetobacter,* increased after DSS treatment. Importantly, XFBD administration further increased the abundance of these symbiotic bacteria (Figures 4C–D’). In summary, the administration of XFBD can restore the imbalance of flies’ intestinal microbiota caused by DSS treatment and increase the number of beneficial bacteria. XFBD partially restored the imbalance of microbial communities caused by DSS by increasing the total microbial count and the proportion of dominant bacterial species, while also enhancing the diversity of microbial communities and promoting the growth of beneficial bacteria.

### 3.5 XFBD inhibited the activation of multiple immune signaling pathways in DSS-induced intestinal disorder

NF-κB signaling pathways exert crucial regulatory roles in intestinal inflammation (Ma et al., 2022). Specifically, in both murine and *Drosophila* models of DSS-induced intestinal inflammation, aberrant hyperactivation of NF-κB pathways serves as a definitive hallmark of inflammatory pathogenesis and constitutes a valid therapeutic target for alleviating intestinal damage (Han et al., 2023; He et al., 2023; Ma et al., 2022; Xiu et al., 2025; Yang et al., 2025). Furthermore, the immune response in fruit fly to intestinal inflammation and gut microbiota dysbiosis is regulated primarily by two NF-κB-related signaling pathways: the IMD pathway and the Toll signaling pathway. The activation of these pathways induces the expression of immune effectors, including antimicrobial peptides (AMPs), thereby efficiently combating infections (Chen et al., 2022).

**Figure 5:**
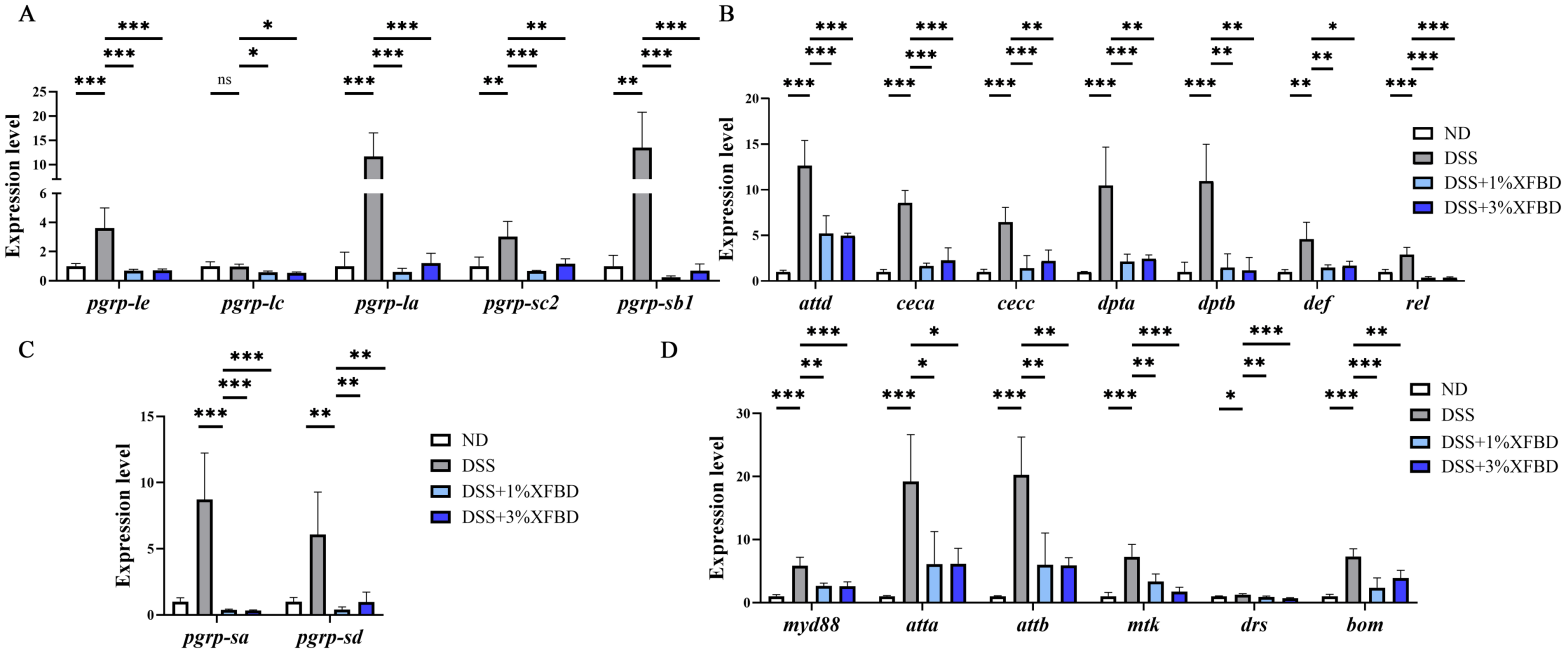
The effects of DSS administration and XFBD treatment on the activation of IMD and Toll immune pathways. **(A)** The expression levels of various PGRPs in the IMD pathway. **(B)** The expression levels of IMD pathway reporter genes. **(C)** The expression levels of various PGRPs in the Toll pathway. **(D)** The expression level of Toll pathway reporter genes (^*^indicates *p* < 0.05; ^**^indicates *p* < 0.01; ^***^indicates *p* < 0.001).

To monitor the alteration of the activity of IMD pathway following DSS or XFBD treatments, we measured the expression levels of various IMD pathway related genes using RT-qPCR, including *peptidoglycan recognition proteins-LE* (*PGRP-LE*), *PGRP-LC*, *PGRP-SB1*, *PGRP-LA*, *PGRP-SC2*, *Relish* (*rel*), as well as the genes encoding IMD targeted AMPs: *Attacin-D* (*attd*), *Cecropin A* (*ceca*), *Cecropin C* (*cecc*), *Diptericin A* (*dpta*), *Diptericin B* (*dptb*), *Defensin* (*def*). DSS treatment increased the expression levels of all these genes except for *PGRP-LC*, suggested that DSS-induced gut inflammation activates the IMD immune response. Conversely, XFBD administration effectively reversed the up-regulation induced by DSS treatment. The expression of *PGRPs*, *rel*, and genes encoding AMPs returned to normal levels, signifying XFBD’s efficient capability to inhibit the IMD pathway activation induced by DSS (Figures 5A,B).

Similar to its impact on the IMD pathway, DSS also induced the activation of the Toll pathway, as evidenced by the upregulation of the expression of these related genes, including *PGRP-SA*, *PGRP-SD*, *Myd88*, and the genes encoding downstream AMPs, such as *Attacin-A* (*atta*), *Attacin-B* (*attb*), *Metchnikowin* (*mtk*), *Drosomycin* (*drs*), and *Bomanin* (*bom*). Conversely, XFBD effectively mitigated this excessive stimulation, restoring the expression levels of these genes to a normal state (Figures 5C,D).

### 3.6 XFBD inhibited the activation of JNK, JAK/STAT pathways in DSS-induced intestinal disorder

In fruit fly, both the c-Jun N-terminal kinase (JNK) pathway and its downstream Janus kinase/signal transducer and activator of transcription (JAK/STAT) pathway are critical for responding to various cellular stresses, regulating the immune system, modulating inflammation, and maintaining stem cell homeostasis (Zhou and Boutros, 2020). It has been reported that these two pathways also play crucial roles in DSS-induced intestinal disorders and also serve as important therapeutic targets for intestinal inflammation (He et al., 2025; Li et al., 2024).

**Figure 6:**
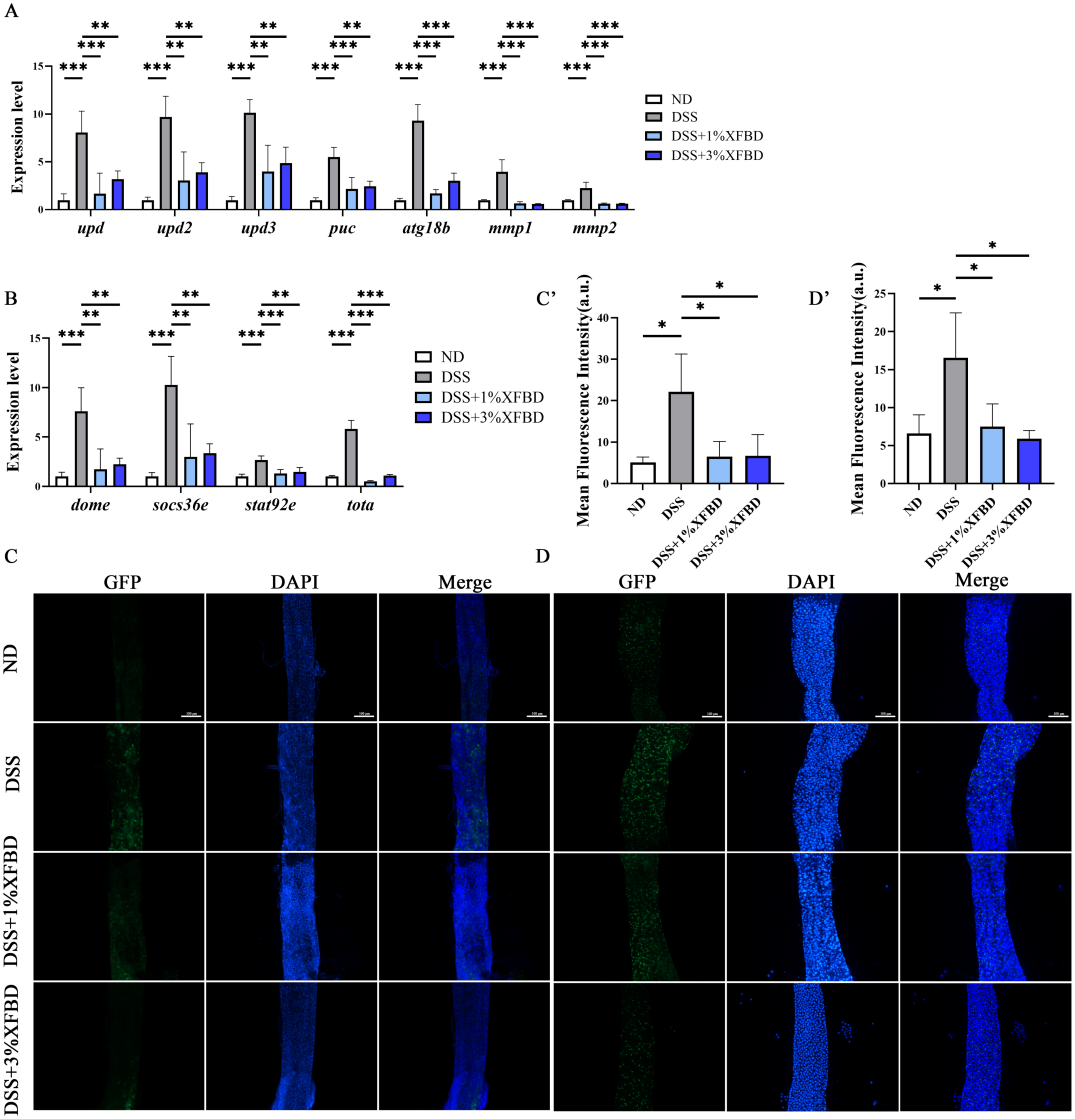
The effects of DSS administration and XFBD treatment on the activation of JNK and JAK/STAT pathways. **(A,B)** The expression levels of JNK-JAK/STAT pathway related genes. **(C)** Fluorescence images of the midguts of upd3-GFP flies within different treatment. Green signal indicated Upd3 expression. DAPI (blue) indicates the location of the nuclei. **(C’)** Mean fluorescence intensity of GFP signal. **(D)** Fluorescence images of the midguts of 10× STAT-GFP flies within different treatment. Green signal indicated activation of STAT. DAPI (blue) indicates the location of the nuclei. **(D’)** Mean fluorescence intensity of GFP signal (^*^indicates *p* < 0.05; ^**^indicates *p* < 0.01; ^***^indicates *p* < 0.001. Scale bar = 100 μm).

Upon introducing DSS, the transcript levels of two JNK targets, *Autophagy-related-18b* (*atg18b*) and *puckered* (*puc*), increased compared to the control group. Concurrently, the expression levels of the downstream target genes, including *upd family cytokines* (*upd1*, *upd2, upd3*), *Matrix metalloproteinase1* (*mmp1*) and *mmp2*, also significantly increased. However, the upregulation of the expression levels of these genes was significantly inhibited after administering XFBD (Figures 6A,B). Furthermore, we visualized cellular Upd3 levels using Upd3-GFP flies, where the production of GFP in the cell reflects the Upd3 level. Consistently, DSS up-regulated Upd3*-GFP* signal in intestinal cells, while XFBD down-regulated it (Figures 6C,C’).

The JAK/STAT pathway exhibited similar responses to DSS and XFBD treatments. The expression levels of *stat92e*, *domeless* (the unique receptor for receiving the signal of Upd cytokines and initiating the JAK/STAT pathway, *dome*), as well as the downstream *suppressor of cytokine signaling protein 36E* (*socs36e*) and *Turandot A* (*totA*), were all upregulated following DSS treatment. Like its impact on the JNK pathway, XFBD effectively countered the impact of DSS on JAK/STAT pathway (Figures 6A,B). Additionally, we also *in situ* confirmed the effects of DSS and XFBD on the activation of JAK/STAT pathway in intestinal cells using special transgenic fly line, which visualizes the *stat* activation with GFP signal (10× STAT GFP) (Figures 6D,D’).

In general, DSS treatment can induce a robust immune and inflammatory response by activating multiple immune and cellular stress pathways. Conversely, XFBD effectively mitigates the effects of DSS by directly inhibiting the activation of these pathways.

### 3.7 Inhibition of IMD and JNK pathways alleviates DSS-induced dysbiosis

**Figure 7:**
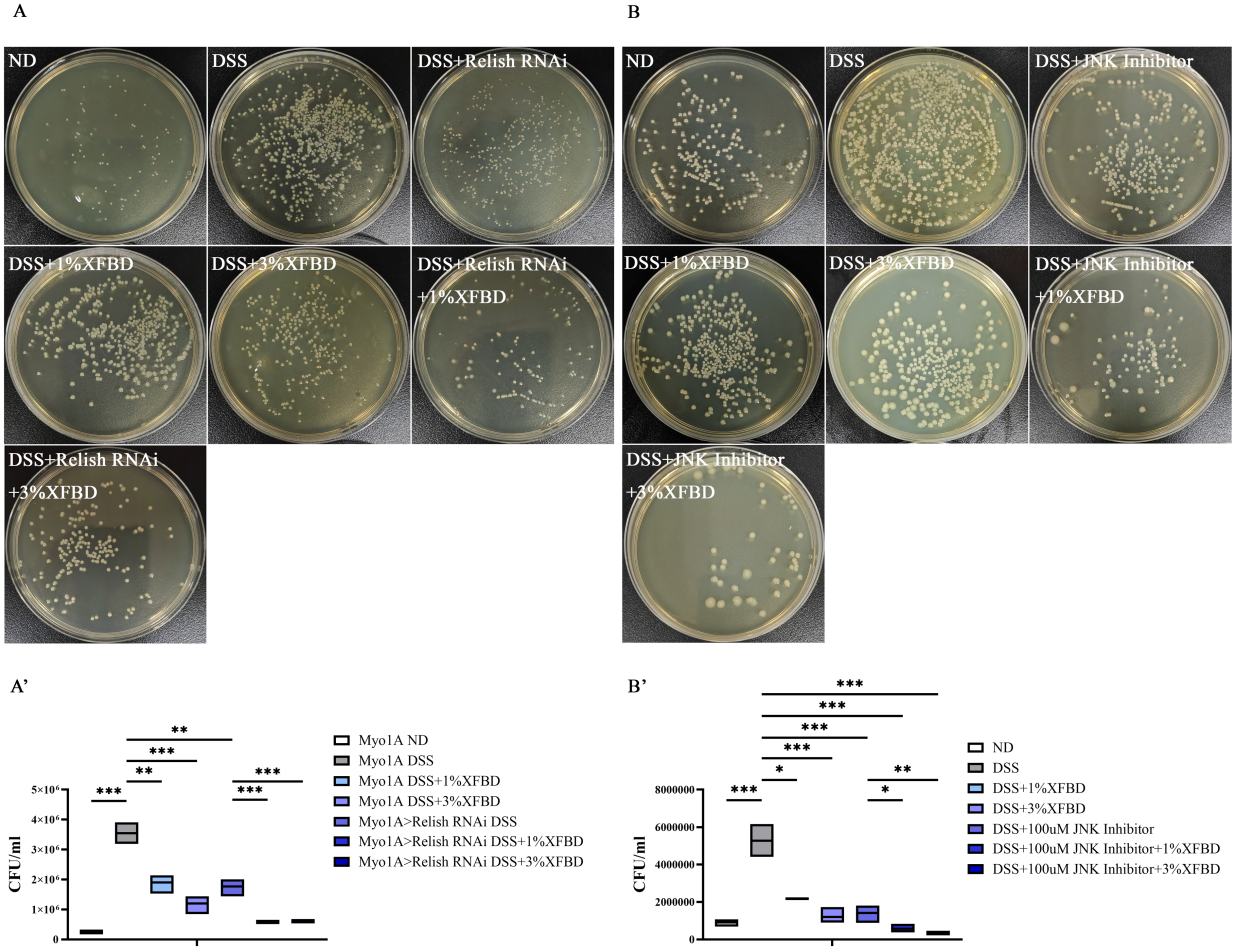
Changes in microbiota following the inhibiting of IMD and JNK pathways. **(A,B)** LB medium cultivation to confirm the relative abundance of the intestinal microbiota of separate groups. **(A’,B’)** CFU number of LB plate (^*^indicates *p* < 0.05; ^**^indicates *p* < 0.01; ^***^indicates *p* < 0.001).

To explore the role of the IMD inhibition effect of XFBD in restoring the homeostasis of fruit fly gut microbiota, we reduced the IMD pathway activity in the intestines by specifically knocking down the expression of *Relish* in midgut enterocytes (ECs) using RNA interference (*Myo1A > Relish RNAi*). The silencing efficiency was evaluated using RT-qPCR. The expression of Relish was reduced by 31% under healthy conditions with a normal diet, and by 80% in the situation of DSS treatment. The expression of Relish is not any more induced by DSS treatment under Relish knockdown, indicating a successful inhibition of the IMD pathway (Supplementary Figure S2). Inhibition of the IMD pathway by silencing *Relish* halted the increase in total microbial counts induced by DSS treatment, leading to a result akin to XFBD administration (Figures 7A,A’). Silencing *Relish* and administering XFBD simultaneously also stopped the increase in total microbial counts induced by DSS. An additive effect was observed: silencing *Relish* and administering XFBD together led to lower microbial counts compared to either intervention alone. This suggests that the restorative effect of XFBD on the gut microbiota is not solely achieved through IMD pathway inhibition.

We also utilized JNK inhibitors (Sp600125) to suppress the JNK pathway, aiming to mimic the JNK inhibiting effect of XFBD and explore its role in restoring microbiota homeostasis. The reductions in the expression of all the JNK target genes of the JNK pathway regardless the treatments indicated the successful blocking of its activation (Figure S3). Like the IMD inhibition. Similarly, JNK inhibition also led a substantial recovery from microbiota disturbance induced by DSS (Figures 7B,B’). An additive effect of JNK inhibition and XFBD treatment was also observed. Collectively, these results indicate that the restorative effect of XFBD on the gut microbiota is more complex than solely inhibiting either the IMD or JNK pathways. Instead, it is achieved through modulating a complex network that involves both IMD and JNK pathways.

### 3.8 Symbiotic microbiota partially attenuates intestinal injury and rescues the premature mortality induced by DSS exposure

We further investigated whether the increased commensal bacteria have exerted a beneficial effect on the gut in fruit flies, particularly in alleviating the impairments induced by DSS.

**Figure 8:**
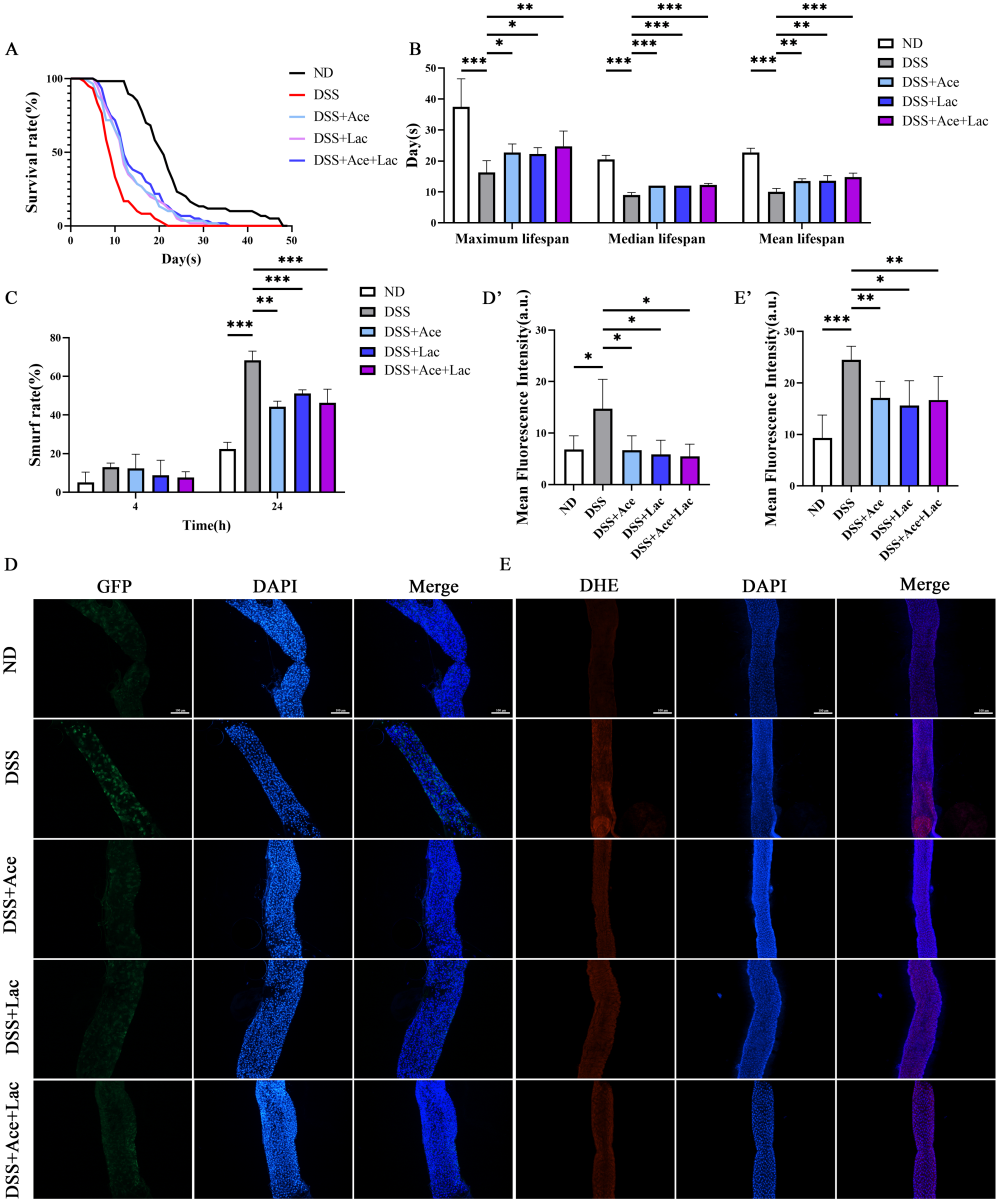
Effect of symbiotic flora replanting on DSS induced intestinal inflammation in *Drosophila melanogaster*. **(A)** Survival curve (*n* = 20) **(B)** The maximum lifespan, median lifespan, and mean lifespan of *Drosophila melanogaster* in diverse groups. **(C)** Effects of symbiotic flora replanting on the DSS induced intestinal integrity of *Drosophila melanogaster* (*n* = 20) **(D)** Fluorescence images of the midguts of ESG-GFP flies within different treatments. **(D′)** Mean fluorescence intensity of GFP signal. **(E)** DHE-staining. **(E’)** Mean fluorescence intensity of DHE signal. (“DSS + Ace” represents wild-type flies that received DSS with Acetobacter. “DSS + Lac” means wild-type flies that received DSS with *Lactobacillus*. “DSS + Ace+Lac” represents wild-type flies that received DSS with *Acetobacter* and *Lactobacillus*. ^*^Indicates *p* < 0.05; ^**^indicates *p* < 0.01; ^***^indicates *p* < 0.001. Scale bar = 100 μm).

We reintroduced *Acetobacter* and *Lactobacillus* separately into the guts of DSS treated fruit flies, resulting in an effectively mitigated the shortened lifespan, intestinal leakage, and excess ROS level in gut caused by DSS treatment (Figures 8A–E’). However, it is noteworthy that the therapeutic effect of reintroducing bacteria was significantly weaker than that of XFBD (Figures 1A–C, 8A–C). This suggests that XFBD’s therapeutic effect is not solely dependent on restoring intestinal microbiota homeostasis but also involves interactions with other factors.

**Figure 9:**
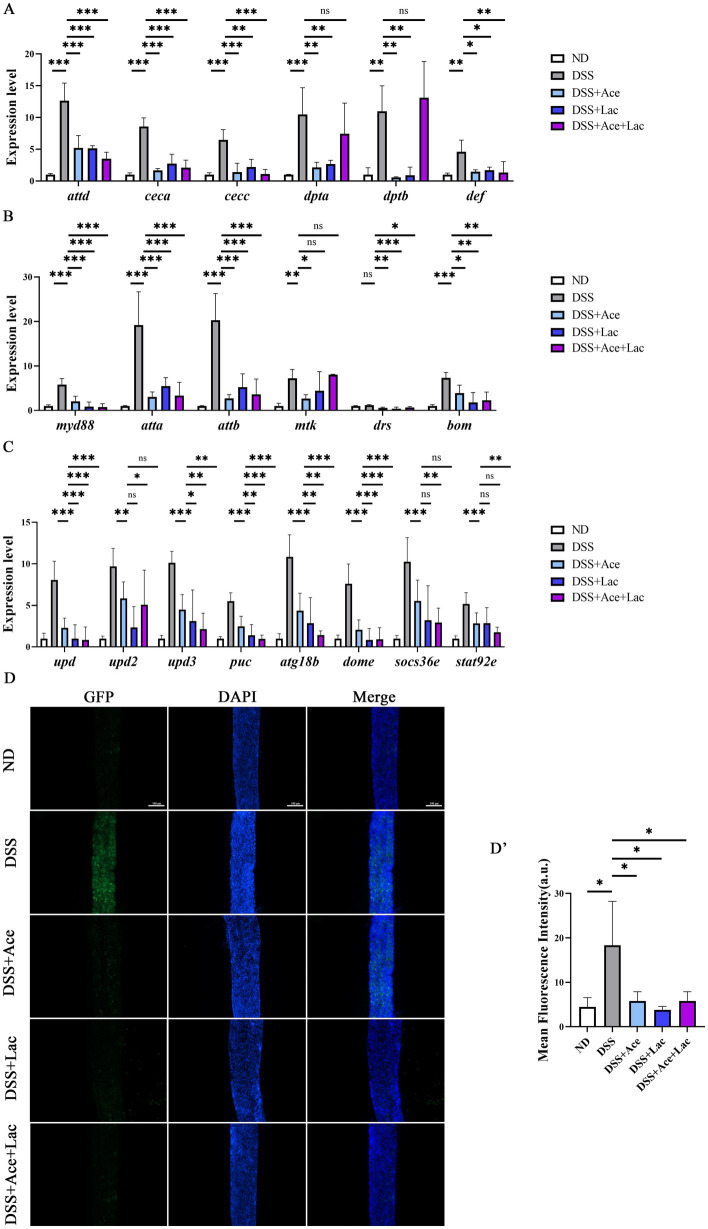
Transcriptional levels of immune pathways, JNK and JAK/STAT pathways after microbe reintroductions. **(A)** The expression levels of IMD pathway related genes. **(B)** The expression levels of Toll pathway related genes. **(C)** The expression levels of JNK and JAK/STAT pathway related genes. **(D)** Fluorescence images of the midguts of upd3-GFP flies within different treatments. **(D’)** Mean fluorescence intensity of GFP signal (^*^indicates *p* < 0.05; ^**^indicates *p* < 0.01; ^***^indicates *p* < 0.001. Scale bar = 100 μm).

For instance, like XFBD, feeding either *Acetobacter* or *Lactobacillus* also resulted in inhibition of division and differentiation of ISCs. Interestingly, reintroducing *Acetobacter* and *Lactobacillus* completely reversed the overactivation of the IMD, Toll, JNK, and JAK/STAT signaling pathways caused by DSS (Figures 9A–D). These findings collectively suggest that the increase in beneficial microbiota following XFBD administration can combat intestinal inflammation, maintain intestinal homeostasis by reducing intestinal ROS levels, and inhibit the excessive activation of immune and inflammatory pathways. This suppression of immune and inflammatory responses further supports the recovery of healthy microbiota, forming a positive feedback loop that enhances the therapeutic activity of XFBD.

These findings collectively suggest that the increase in beneficial microbiota following XFBD administration can combat intestinal inflammation, maintain intestinal homeostasis by reducing intestinal ROS levels, and inhibit the excessive activation of immune and inflammatory pathways. This suppression of immune and inflammatory responses further supports the recovery of healthy microbiota, forming a positive feedback loop that enhances the therapeutic activity of XFBD.

## 4 Discussion and conclusion

XFBD is formulated by Academician Boli Zhang and Professor Qingquan Yu for the treatment of COVID-19. Clinical studies have shown that XFBD combined with antiviral drugs can accelerate the virus clearance rate and shorten the hospital stay (Huang et al., 2021).

Previous studies have reported that, XFBD inhibits the pro-inflammatory response to lung disease (L. Ma et al., 2022). Bi et al. (2022) have documented XFBD’s ability to alleviate intestinal symptoms such as diarrhea and constipation in patients, thereby preventing the further progression of the disease. (Ma et al. (2022) established a mouse intestinal inflammation model induced by DSS to simulate intestinal diseases caused by COVID-19. They found that XFBD significantly blocks NF-κB signaling pathway, reshapes intestinal immunity and modifies microbiota. This leads to a reduction in COVID-19 related intestinal inflammation.

In clinical practice, the “Lung–Gut Axis” is a critical framework for understanding the coexistence of respiratory and gastrointestinal (GI) diseases. The mechanism underlying GI symptoms in COVID-19 patients—including those treated clinically with XFBD—is primarily associated with the dysregulation of ACE2 expression, which leads to damage to the intestinal mucosal barrier, dysregulation of intestinal flora, and a systemic elevation of inflammatory factors (often termed a “cytokine storm”). Concurrently, the characteristic of DSS-induced enteritis involves a compromised mucosal immune response triggered by microbial exposure (Kim et al., 2012). Moreover, DSS treatment disrupts the inflammatory response in the intestine, leading to damage to the intestinal barrier and the manifestation of symptoms associated with intestinal inflammation, such as oxidative stress response and immune dysfunction (Chassaing et al., 2014; Lima et al., 2019). In this work, we established an intestinal inflammation model on fruit flies by DSS administration. Like the mouse model, DSS administration in fruit fly led to a shortened lifespan, intestinal damage, oxidative stress of fruit flies, and accompanied by microbial community imbalance. This model thus provides a robust platform to investigate how XFBD mitigates GI distress by restoring the intestinal barrier and microbial homeostasis, thereby bridging clinical observations with mechanistic insights.

In *Drosophila*, the IMD and Toll pathways represent two major upstream signaling cascades that regulate the NF-κB pathway and mediate humoral. Gram-negative bacteria activate the IMD pathway to trigger the NF-κB-like transcription factor Relish and induce antimicrobial peptide (AMP) production (Chen et al., 2022). Meanwhile, Gram-positive bacteria and fungi activate the Toll-NF-κB pathway via PGRP/GNBP-mediated signaling to engage MyD88 and the NF-κB homologous Dorsal-Dif complex, and DSS administration similarly activates this pathway by increasing the expression of PGRP-SA/SD, myd88, and corresponding AMPs (Lindberg et al., 2018). DSS-induced intestinal inflammation activates the NF-κB-dependent IMD pathway (via upregulating PGRP-LA/LE, rel, AMPs, and IMD-controlled negative regulators PGRP-SB1/SC2) and the Toll pathway (via upregulating PGRP-SA/SD, myd88, and corresponding AMPs); however, XFBD inhibits both pathways to normalize related gene transcription, and Relish knockdown mimics XFBD’s IMD-inhibitory effect, restoring bacterial populations and indicating that regulating these pathways may maintain microbiota homeostasis.

In another study, it was showed that XFBD can effectively treat Bleomycin-induced COPD in mice by inhibiting the activation of IL-6/STAT3 pathway (Wang et al., 2022). In fruit flies, the JNK signaling pathway plays a crucial role in sensing changes in ROS levels, DNA damage, and bacterial infections within the organism (Tian et al., 2015). Under stress condition, the JNK pathway is actived, in turn, promotes the expression of target genes, including *mmp1/2*, *upd family cytokines*, and a negative feedback regulator of JNK, *puc*, to modulate various processes (Xu et al., 2017). The Upd family cytokines are homologous of IL-6, and they further stimulate the JAK/STAT pathway (Yu et al., 2022). Consistent with results from the mouse model, the DSS administration activated JNK-Upd3-JAK/STAT pathway axis in fruit fly, while XFBD effectively inhibited the over-activation of both the JNK and JAK/STAT pathway. Moreover, the JNK pathway, which collaborates with the IMD pathway to produce AMPs, and the JAK/STAT pathway are also involved in the immune regulation process in both fruit flies and mammals. Subsequently, we used JNK inhibitors to mimic XFBD’s inhibitory effect on JNK pathway and found that the regulation of the JNK pathway by XFBD could be another potential approach for restoring microbiota homeostasis.

We also found that the administration of XFBD could significantly inhibit the disruption of intestinal integrity induced by DSS, and this protective effect is primarily attributed to its suppression of excessive JNK pathway activation. In the context of intestinal inflammation triggered by DSS, JNK pathway hyperactivation is a key driver of intestinal barrier impairment, acting through two distinct mechanisms. On the one hand, the JNK pathway directly upregulates the expression of its downstream target genes *mmp1* and *mmp2* (Yu et al., 2022). As an enzyme responsible for cleaving extracellular matrix (ECM) components, Mmp2 promotes ECM degradation, which in turn leads to intestinal barrier dysfunction (Zhou and Boutros, 2020). On the other hand, JNK pathway activation mediates the hyperactivation of the JAK/STAT pathway, thereby inducing aberrant proliferation of midgut stem cells and disrupting the normal architecture of intestinal epithelial cells.

Consistent with this mechanistic framework, our results demonstrated that XFBD treatment not only effectively alleviated the excessive activation of the JNK pathway and markedly reduced the expression levels of *mmp1* and *mmp2* to preserve ECM integrity, but also blocked the abnormal activation of the JAK/STAT pathway downstream of JNK, ultimately restoring the balance of midgut cell renewal.

The reintroduction of symbiotic bacteria also has a rescuing effect on the shortened lifespan resulting from DSS exposure. However, the effect is weaker compared to the effect of XFBD. The simultaneous reintroduction of multiple beneficial symbiotic bacteria may have a more potent therapeutic impact on intestinal inflammation and dysbiosis in fruit flies, warranting further investigation. Additionally, XFBD therapy not only restores the balance of the intestinal microflora but also addresses intestinal inflammation, leading to an extension of lifespan through a combination of multiple mechanisms. The microbial community structure of the XFBD group does not overlap with those of either the control group or the DSS model group in the PCA results. XFBD not only enhanced the richness and diversity of the gut microbiota in fruit fly compared to the control and DSS model groups but also regulated the abundance of symbiotic microbiota. The altered microbial composition may contribute to its efficacy in increasing lifespan, even surpassing that of the flies in the control group.

XFBD contains numerous active substances, such as quercetin, luteolin, stigmasterol, and kaempferol, which exhibit with anti-inflammatory and antioxidant activities (Yan et al., 2021; Zhao et al., 2023). These components have demonstrated therapeutic effects on various diseases. This is evident in their inhibitory effects on the JNK pathway, nuclear factor-κB (NF-κB) pathway, and inflammation, which align with our experimental results (Gao et al., 2021; Jiang et al., 2022; Wu et al., 2017; Xiao et al., 2023). Our findings have also shown that XFBD effectively inhibits the activation of inflammation related JNK, JAK/STAT and NF-κB pathways by DSS and reduces intestinal ROS levels in gut. The inhibition of multiple pathways by XFBD also contributes to a certain restoration of the dysbiosis of the gut microbiota in fruit flies. The increase in symbiotic microbiota can further mitigate the elevated ROS levels and excessive pathway activation in fruit flies, creating a positive cycle and exerting a therapeutic effect on the intestinal inflammation caused by DSS. Simultaneously, XFBD inhibitors the proliferation of intestinal stem cells and enhances intestinal integrity through a JAK/STAT independent pathway. These various effects of XFBD may collectively contribute to its ability to extend lifespan and protect against DSS-induced intestinal inflammation.

In summary, our findings demonstrate that XFBD effectively alleviates DSS-induced intestinal inflammation, resulting in a significant extension of the flies’ lifespan by regulating intestinal microbiota and suppressing inflammation through the modulation of immune homeostasis, particularly by inhibiting the JNK, JAK/STAT, and NF-κB pathways. Further research is warranted to comprehensively understand the specific roles and detailed mechanisms underlying these effects of XFBD. Such insights will be invaluable for the prevention and treatment of gut and lung diseases using XFBD.

A key limitation of the present study is its exclusive use of the *Drosophila* model: although key inflammatory pathways (NF-κB, JNK, JAK/STAT) are evolutionarily conserved between fruit flies and mammals, their intestinal anatomy, immune signaling cascades, and microbiota composition differ substantially. Thus, the therapeutic efficacy and mechanistic interpretations of XFBD identified here need validation in mammalian models (e.g., mice) to enhance clinical translational relevance.

## Data Availability

The datasets presented in this study can be found in online repositories. The names of the repository/repositories and accession number(s) can be found at: https://www.ncbi.nlm.nih.gov/, PRJNA1387016.

## Glossary

ACE2: angiotensin-converting enzyme 2
AMP: antimicrobial peptide
Atg18b: Autophagy-related-18b
AttA: Attacin-A
AttB: Attacin-B
AttD: Attacin-D
Bom: Bomanin
CecA: Cecropin A
CecC: Cecropin C
CFU: colony-forming units
COVID-19: coronavirus disease 2019
DptA: Diptericin A
DptB: Diptericin B
Def: Defensin
DHE: dihydroethidium
Dome: domeless
Drs: Drosomycin
DSS: dextran sulfate sodium
EC: enterocyte
ECM: extracellular matrix
GFP: green fluorescent protein
GNBP: glucan binding protein
IMD: immunodeficiency
ISC: intestinal stem cell
Mmp: Matrix metalloproteinase
Mtk: Metchnikowin
NF-κB: nuclear factor-κB
PACS: post-acute COVID-19 syndrome
PBS: phosphate-buffered saline
PCA: principal component analysis
PGN: peptidoglycan
PGRP: peptidoglycan recognition proteins
Puc: puckered
RBD: receptor-binding domain
Rel: Relish
ROS: reactive oxygen species
Socs36E: suppressor of cytokine signaling at 36E
TCM: Traditional Chinese Medicine
TMPRSS2: transmembrane protease serine 2
Tot: Turandot
Upds: unpaireds
Upd2: unpaired 2
Upd3: cytokine unpaired 3
XFBD: Xuanfei Baidu decoction
