# Supplementary material for: Xuanfei Baidu decoction alleviates intestinal inflammation and modulates microbiota distribution by suppressing IMD/NF-κB and JNK signaling pathways
Source: Front Microbiol. 2026 Jun 10;17:1853715. doi: 10.3389/fmicb.2026.1853715 (PMC13291020; doi:10.3389/fmicb.2026.1853715)
Supplement: Supplementary file 1 [file Data_Sheet_1.docx]

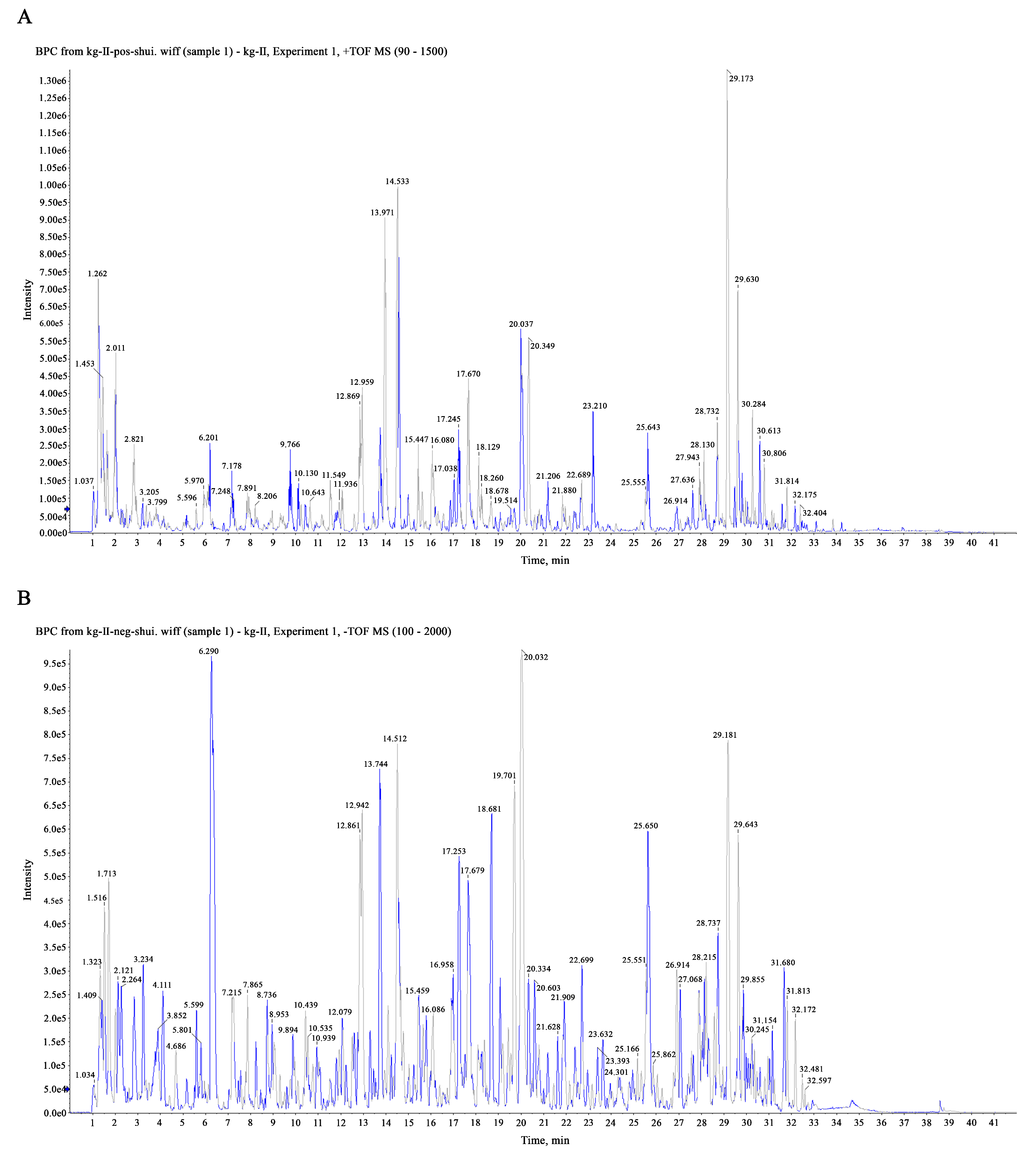
Figure S1. Base peak mass spectrum of XFBD with positive and negative modes.


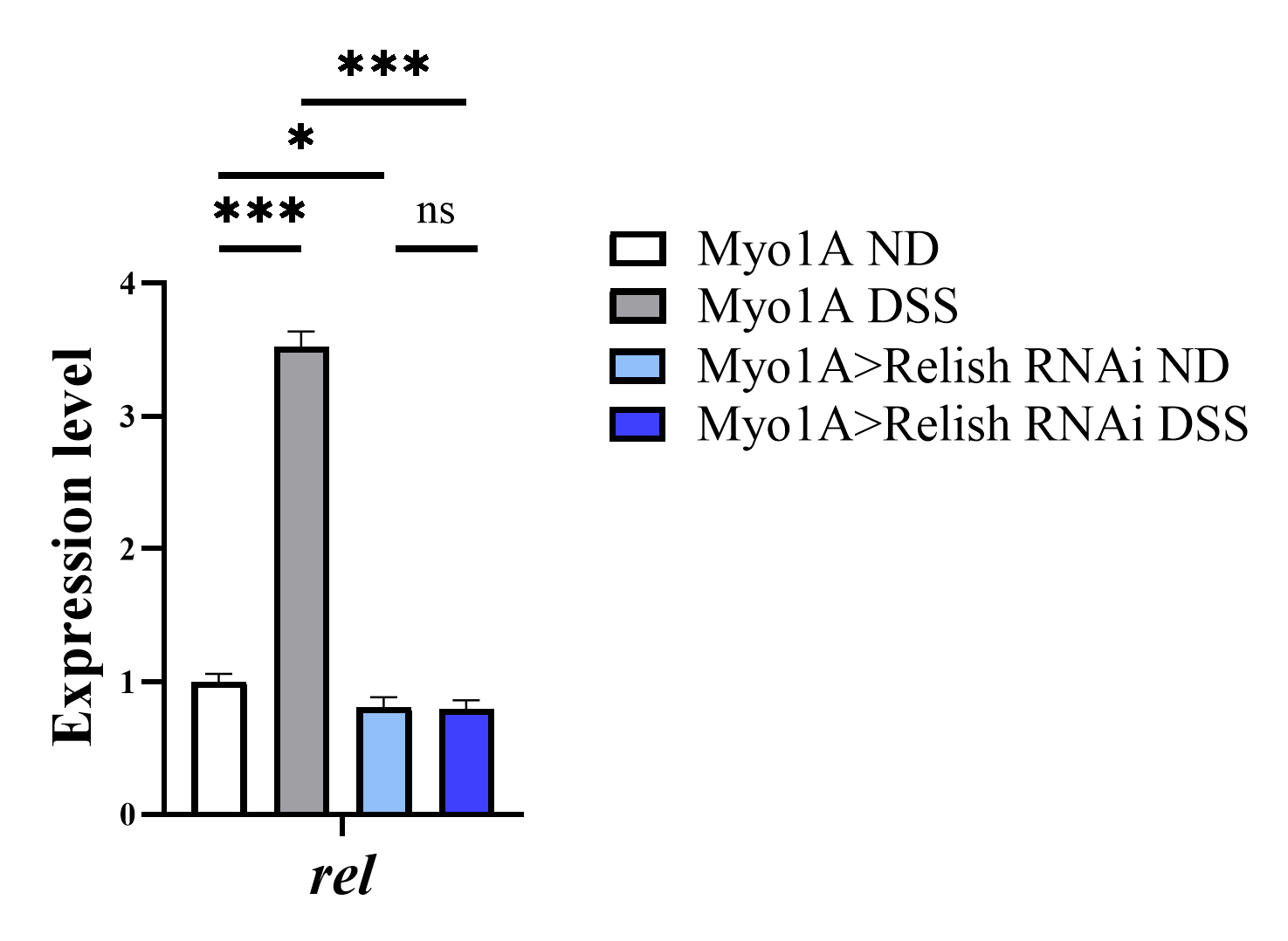
Figure S2. The qPCR results of relish silencing efficiency in the Myo1A>Relish RNAi. (* indicates *P* < 0.05; * * indicates *P* < 0.01; * * * indicates *P* < 0.001.)


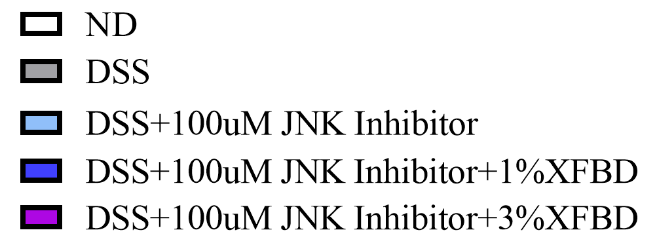

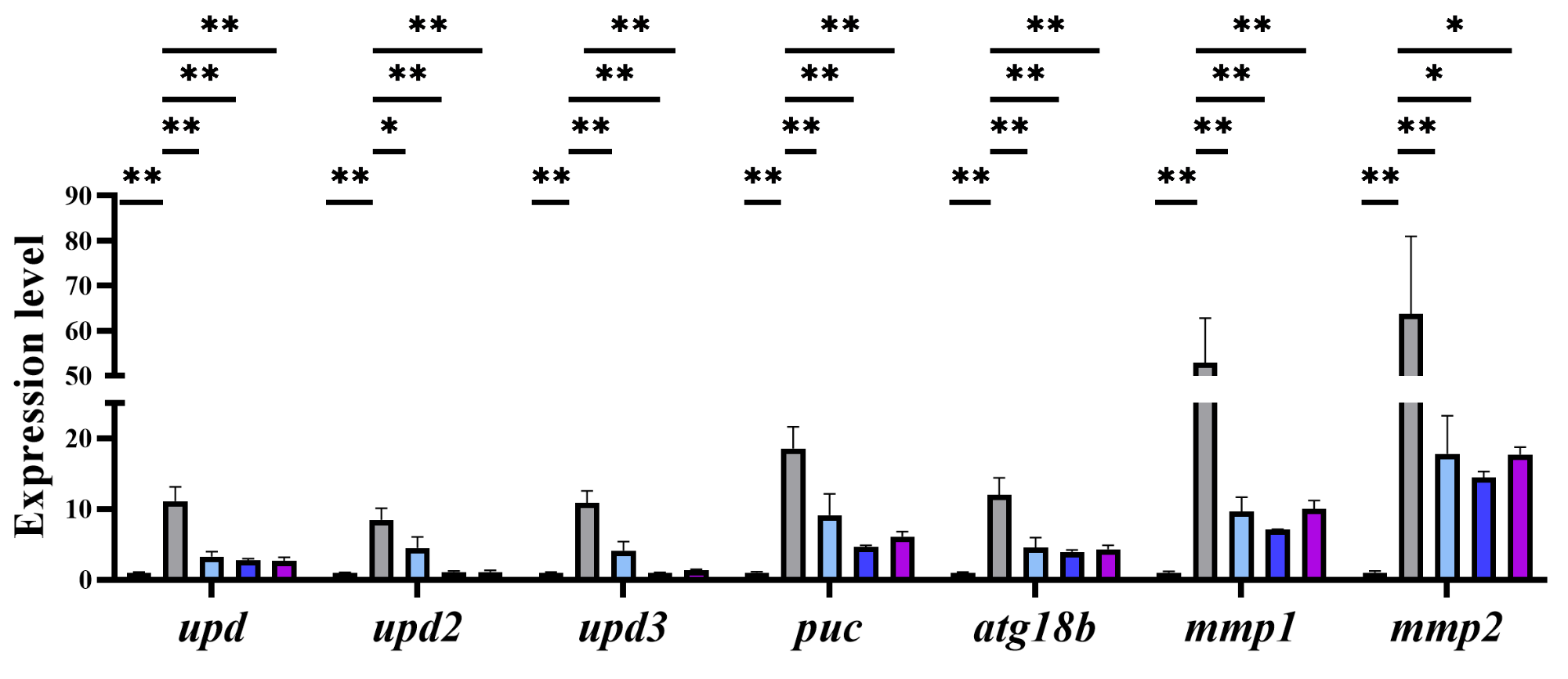


Figure S3. The qPCR results of JNK pathway related genes after JNK inhibitor treatment. (* indicates *P* < 0.05; * * indicates *P* < 0.01; * * * indicates *P* < 0.001.)

**Table S1. Compound list for XFBD identified by UPLC-Q-TOF/MS.**

| **No.** | **RT**  **(min)** | **Negative ion** | **Positive ion** | **Molecular Formula** | **Identity** | **source** |
| --- | --- | --- | --- | --- | --- | --- |
| **1** | 1.23 |  | 175.1189 | C_6_H_14_N_4_O_2_ | L-Arginine |  |
| **2** | 1.41 | 341.1095 |  | C_12_H_22_O_11_ | Maltose/Sucrose |  |
| **3** | 1.45 |  | 266.1243 |  | D-1-[(3-Carboxypropyl)amino]-1-deoxyfructose |  |
| **4** | 1.51 | 191.0565 |  | C_7_H_12_O_6_ | Quinic acid |  |
| **5** | 1.7 | 341.1093 |  | C_12_H_22_O_11_ | Maltose/Sucrose | MBC |
| **6** | 1.99 | 711.2218 |  | C_24_H_42_O_21_ | Stachyose | TLZ |
| **7** | 3.89 | 287.0767 |  | C_12_H_16_O_8_ | 1-β-D-glucopyranosyloxy-3,5-dihydroxybenzene |  |
| **8** | 5.6 |  | 346.0563 | C_10_H_12_N_5_O_7_P | Cyclic guanosine monophosphate |  |
| **9** | 6.19 |  | 268.1037 | C_10_H_13_N_5_O_4_ | Adenosine* | LG |
| **10** | 9.76 |  | 166.1231 | C_10_H_15_NO | Ephedrine * | MH |
| **11** | 9.88 | 353.0896 |  | C_16_H_18_O_9_ | 5-O-Caffeoylquinic acid* | GHX/KXR |
| **12** | 10.54 | 461.1671 |  | C_20_H_30_O_12_ | Verbasoside |  |
| **13** | 10.65 |  | 180.1396 | C_11_H_17_NO | N-Methylephedrine | MH |
| **14** | 11.81 | 289.0718 |  | C_15_H_14_O_6_ | (+)-Catechin* | MH/KXR/CZ/HZ |
| **15** | 11.94 | 787.196 | 789.2103 | C_33_H_40_O_22_ | Quercetin-3-O-β-D-glucose-7-O-β-D-gentiobioside* | TLZ |
| **16** | 11.94 |  | 627.1584 | C_27_H_30_O_17_ | Quercetin 7-gentiobioside | TLZ |
| **17** | 12.1 | 353.0882 |  | C_16_H_18_O_9_ | Chlorogenic acid* |  |
| **18** | 12.51 | 353.0879 |  | C_16_H_18_O_9_ | 4-O-Caffeoylquinic acid* |  |
| **19** | 12.61 |  | 193.0506 | C_10_H_8_O_4_ | Scopoletin or isomer | LG |
| **20** | 12.75 | 367.1037 |  | C_17_H_20_O_9_ | 3-O-Feruloylquinic acid |  |
| **21** | 12.95 | 456.1526 | 458.1668 | C_20_H_27_NO_11_ | Amygdalin* | KXR |
| **22** | 12.99 | 365.1447 |  | C_15_H_26_O_10_ | 3,4-Dihydroharpagide |  |
| **23** | 13.73 | 403.1251 | 405.14 | C_17_H_24_O_11_ | Hastatoside* | MBC |
| **24** | 13.75 |  | 207.0652 | C_11_H_10_O_4_ | Scoparone | QH |
| **25** | 13.77 |  | 225.0761 | C_11_H_12_O_5_ | Sinapinic acid | TLZ |
| **26** | 14.11 | 435.1512 | 408.1874 | C_17_H_26_O_10_ | 3,4-Dihydroverbenalin | MBC |
| **27** | 14.5 |  | 195.066 | C_10_H_10_O_4_ | Ferulic acid or isomer |  |
| **28** | 14.5 |  | 357.1185 | C_16_H_20_O_9_ | Gentiopicroside | MBC |
| **29** | 14.52 | 387.1291 | 389.1454 | C_17_H_24_O_10_ | Verbenalin* | MBC |
| **30** | 14.59 | 593.1541 | 595.1669 | C_27_H_30_O_15_ | Vicenin-2 | GC |
| **31** | 15.46 | 367.1039 |  | C_17_H_20_O_9_ | 5-O-ferruloylquinic acid |  |
| **32** | 16.07 | 563.143 | 565.1576 | C_26_H_28_O_14_ | Schaftoside* | GC |
| **33** | 16.21 |  | 479.0831 | C_21_H_18_O_13_ | Quercetin 3-O-β-D-glucuronopyranoside | HZ |
| **34** | 17.04 | 621.1113 | 623.126 | C_27_H_26_O_17_ | Apigenin 7-diglucuronide |  |
| **35** | 17.22 |  | 193.05 | C_10_H_8_O_4_ | Scopoletin or isomer | LG |
| **36** | 17.25 | 389.1255 |  | C_20_H_22_O_8_ | Polydatin* | HZ |
| **37** | 17.64 | 417.1207 |  | C_21_H_22_O_9_ | Liquiritin or isomer | GC |
| **38** | 17.65 | 549.1631 |  | C_26_H_30_O_13_ | Licuraside or isomer | GC |
| **39** | 17.67 |  | 257.0816 | C_15_H_12_O_4_ | Isoliquiritigenin / Pinocembrin | GC/KXR/ QH |
| **40** | 18.68 |  | 495.114 | C_22_H_22_O_13_ | 3,5,7,3',4'-pentahydroxy-6-methoxyflavone-3-O-β-D-glucopyranoside |  |
| **41** | 18.7 | 623.2013 | 625.2149 | C_29_H_36_O_15_ | Acteoside* | MBC/GHX/KXR |
| **42** | 19.08 | 515.1218 |  | C_25_H_24_O_12_ | 3,4-Dicaffeoylquinic acid* |  |
| **43** | 19.44 | 541.1373 | 543.1515 | C_27_H_26_O_12_ | Galloyl piceid | HZ |
| **44** | 19.54 | 515.1208 |  | C_25_H_24_O_12_ | 3,5-dicaffeoylquinic acid* |  |
| **45** | 19.71 | 623.1997 | 625.214 | C_29_H_36_O_15_ | Isoacteoside* | MBC |
| **46** | 20.03 | 579.1732 | 581.1884 | C_27_H_32_O_14_ | Naringin* | HJH/HZ/GC |
| **47** | 20.05 |  | 273.0766 | C_15_H_12_O_5_ | Naringenin | HJH/HZ/GC |
| **48** | 20.12 |  | 595.1676 | C_27_H_30_O_15_ | Nicotiflorin | GC |
| **49** | 20.34 | 577.1586 | 579.1729 | C_27_H_30_O_14_ | Rhoifolin | HJH |
| **50** | 20.38 |  | 447.0933 | C_21_H_18_O_11_ | Apigenin-7-O-glucuronide |  |
| **51** | 20.59 | 515.1212 |  | C_25_H_24_O_12_ | 4,5-Dicaffeoylquinic acid* |  |
| **52** | 20.75 | 609.1854 | 611.1998 | C_28_H_34_O_15_ | Hesperidin* | MH |
| **53** | 20.8 | 445.1147 |  | C_22_H_22_O_10_ | Tilianine | MH |
| **54** | 20.82 |  | 285.0767 | C_16_H_12_O_5_ | Physcion | HZ |
| **55** | 20.83 | 359.0774 |  | C_18_H_16_O_8_ | Rosmarinic acid* | MH |
| **56** | 21.85 | 549.1634 | 551.1774 | C_26_H_30_O_13_ | Isoliquiritin apioside* | GC |
| **57** | 21.92 | 431.0989 |  | C_21_H_20_O_10_ | Cosmetin | MH |
| **58** | 22.4 | 417.1196 | 419.1352 | C_21_H_22_O_9_ | Isoliquiritin* | GC |
| **59** | 22.65 |  | 431.1359 | C_22_H_22_O_9_ | Ononin* | GC |
| **60** | 23.4 | 529.136 |  | C_26_H_26_O_12_ | 3,5-di-O-caffeoyl-quinic acid methyl ester or isomer |  |
| **61** | 24.53 | 251.1297 |  | C_15_H_10_O_6_ | Luteolin* | MH |
| **62** | 25.55 | 407.1351 |  | C_20_H_24_O_9_ | Marmesinin or isomer | MBC |
| **63** | 25.63 | 431.0994 |  | C_21_H_20_O_10_ | Cosmetin or isomer | MH |
| **64** | 26.93 |  | 265.1441 | C_15_H_20_O_4_ | Vulgarin | KXR |
| **65** | 27.07 | 517.1008 |  | C_24_H_22_O_13_ | 6''-O-malonylgenistin or isomer |  |
| **66** | 27.29 |  | 271.0616 | C_15_H_10_O_5_ | Apigenin* | MH |
| **67** | 27.96 | 445.1149 |  | C_22_H_22_O_10_ | Physcion 1-glucoside | HZ |
| **68** | 28.07 | 371.1509 |  | C_21_H_24_O_6_ | Isoarctigenin / Arctigenin | KXR |
| **69** | 28.13 |  | 469.3318 | C_30_H_44_O_4_ | Glabrolide | GC |
| **70** | 28.21 | 265.1456 |  | C_15_H_22_O_4_ | Magnograndiolide or isomer | QH/ |
| **71** | 28.36 |  | 355.1546 | C_21_H_22_O_5_ | Notopterol | MBC/ GC/ |
| **72** | 28.59 | 265.1455 |  | C_15_H_22_O_4_ | Magnograndiolide or isomer | GHX |
| **73** | 28.71 | 359.0787 | 361.0927 | C_18_H_16_O_8_ | Eupatin | QH |
| **74** | 28.76 |  | 469.3323 | C_30_H_44_O_4_ | Glabrolide or isomer | GC |
| **75** | 28.76 |  | 487.3418 | C_30_H_46_O_5_ | Glabric acid | GC |
| **76** | 29.16 |  | 471.3485 | C_30_H_46_O_4_ | Glycyrrhetic acid | GC |
| **77** | 29.18 | 821.4006 | 823.4155 | C_42_H_62_O_16_ | Glycyrrhizic acid* | GC |
| **78** | 29.69 |  | 471.2021 | C_26_H_30_O_8_ | Limonin | HJH/CZ/HZ/GHX |
| **79** | 29.87 | 531.2241 | 533.2388 | C_28_H_36_O_10_ | Nomilinoate A-ring lactone | HJH |
| **80** | 29.99 |  | 825.4291 | C_42_H_64_O_16_ | Uralsaponin C | GC |
| **81** | 30.4 |  | 515.2291 | C_28_H_34_O_9_ | Nomilin | HZ |
| **82** | 30.47 | 355.1195 |  | C_20_H_20_O_6_ | 5'-prenyleriodictyol | GC |
| **83** | 30.61 |  | 231.1382 | C_15_H_18_O_2_ | Atractylenolide I | CZ |
| **84** | 30.92 |  | 249.1481 | C_15_H_20_O_3_ | Atractylenolide Ⅲ* | CZ |
| **85** | 31.14 |  | 345.0979 | C_18_H_16_O_7_ | Penduletin | LG |
| **86** | 31.15 | 249.1504 |  | C_15_H_22_O_3_ | Qinghaosu V | QH |
| **87** | 31.24 | 337.1454 | 339.1607 | C_21_H_22_O_4_ | Licochalcone C | GC |
| **88** | 31.68 | 269.0464 | 271.0594 | C_15_H_10_O_5_ | Emodin* | HZ |
| **89** | 31.81 | 335.0936 | 337.1078 | C_20_H_16_O_5_ | Glabrone | GC |
| **90** | 32.18 | 351.0884 | 353.1032 | C_20_H_16_O_6_ | Semilicoisoflavone B | GC |
| **91** | 32.65 |  | 225.1126 | C_12_H_16_O_4_ | Pogostone* | GHX |

* Identified with reference standard.

**Table S2. List of qPCR primers**

| Gene | Forward | Reverse |
| --- | --- | --- |
| *PGRP-LE* | TATGTGCCGCAATCTGTTGG | GGTGCTCTTCTTTTGTCCCTCA |
| *PGRP-LC* | CGCAGGGTATTGGCAGCAT | GCCGGATCTTCGTGTTTGG |
| *PGRP-LA* | GTTGGGAGCAAGCGGTTTAT | GGGGTACTGGTATTGGCACTG |
| *PGRP-SC2* | GGGTGCTCACGCCACTAACT | TGCCGATGTCCGTACAGGAT |
| *PGRP-SB* | CAGATTGAACCACGCAGCAG | TGGAGCAGCCATTAGGATTG |
| *PGRP-SA* | AGGCACTGGTTGGGGATTG | CGGGACTCTGGGTGCTGAT |
| *PGRP-SD* | CCCTGGGCATTGCGTTTAT | GGACTCTTGGTAGCACTGACTTGAC |
| *AttA* | TACTCCCACATCAACGGACA | TCCCGTGAGATCCAAGGTAG |
| *AttB* | CTTCGAGTTCCAGCGCAATG | GGTCCAAGGTAGTAGCACGA |
| *AttD* | GTCACTAGGGTTCCTCAG | GCCGAAATCGGACTTG |
| *CecA* | TCTTCGTTTTCGTCGCTCTCA | ATTCCCAGTCCCTGGATTGTG |
| *CecC* | TCATCCTGGCCATCAGCATT | CGCAATTCCCAGTCCTTGAAT |
| *DptA* | GCTGCGCAATCGCTTCTACT | TGGTGGAGTGGGCTTCATG |
| *DptB* | AGCCTGAACCACTGGCATA | AGATCGAATCCTTGCTTTGG |
| *Def* | GAGCCACATGCGACCTACTC | CAGTAGCCGCCTTTGAACC |
| *Relish* | TACAAGAGCGAGATGCATGG | ATAAATTGCGCCACATAGCC |
| *Myd88* | ATCTCGGTGGCATGATCGAT | CTGCTTCGAGACCAACAACA |
| *Mtk* | CCACCGAGCTAAGATGCAA | GCTCTGCCAGCACTGATGTA |
| *Drs* | ACCAAGCTCCGTGAGAACCTT | TTGTATCTTCCGGACAGGCAG |
| *Bomanin* | AGCTCCTAGATATGCGCCAG | CGCAGCCGGAAATGTGATTA |
| *upd* | CCACGTAAGTTTGCATGTTG | CTAAACAGTAGCCAGGACTC |
| *upd2* | AGTGCGGTGAAGCTAAAGACTTG | GCCCGTCCCAGATATGAGAA |
| *upd3* | ACAGATTCCTGCCCCGTCT | GGTCGCGATGGGCGT |
| *puc* | CACCCATCCCAAACACCATG | CCCTCGTCAAATTGCTAGCC |
| *Atg18b* | ATCCGATGCAAGACAACAGC | GTTGGAGTGTCGATGGCATC |
| *Mmp1* | TGGTTTGTTTTGCGCCCATA | GAGATGCCCACTTTGACGAC |
| *Mmp2* | TACTTGTGGCGCATTGGAAC | ATCGATGTGGGTCAAAGTGG |
| *Dome* | TCTGTCTGTGAACGCCACTC | TATCGAACCCTCGCATTCCG |
| *socs36E* | ATGGGTCATCACCTTAGCAAGT | TCCAGGCTGATCGTCTCTACT |
| *Stat92E* | TAGTCCAGTGTGGTGGAATTCGCCACCATGCCGCTAAATCCCTACAACATGAAC | ATCATGTCTGGATCCCTCGAGTCAAAAGTTCTCAAAGTTTGTA ATCGTATCGAAGTCC |
| *totA* | TGCTGCTGATTAGTCCTCTATG | GATGTCAAGTAGTTCCTGGGTG |
| *Rp49* | ATCGGTTACGGATCGAACAAGC | GTAAACGCGGTTCTGCATGAGC |
